# Hsa_circ_0000313/miR‐1224‐3p/MKNK2 Axis Modulates CD4^+^ T Cells by Activating p38 MAPK Signaling in Myasthenia Gravis

**DOI:** 10.1155/mi/2877539

**Published:** 2026-03-20

**Authors:** Ying Li, Xiaotong Kong, Hanlu Cai, Wenqi Tian, Yingjie Ren, Fanfan Xu, Shanshan Peng, Jingyan Niu, Guanghao Xin, Jianjian Wang, Huixue Zhang, Lihua Wang

**Affiliations:** ^1^ Department of Neurology, The Second Affiliated Hospital of Harbin Medical University, Harbin, Heilongjiang, 15000, China, hrbmush.edu.cn

**Keywords:** hsa_circ_0000313, miR-1224-3p, MKNK2, myasthenia gravis (MG)

## Abstract

**Background:**

Myasthenia gravis (MG) is an autoimmune disorder in which circular RNAs (circRNAs) are increasingly implicated, with growing evidence supporting their critical role in autoimmune pathogenesis. The role of hsa_circ_0000313 in MG, including its biological functions and mechanisms, remains unknown.

**Methods:**

Agarose gel electrophoresis, RNase R digestion, and Sanger sequencing were employed to verify the circular structure of hsa_circ_0000313, while nucleoplasmic separation experiment was used to determine its subcellular localization. Analysis of hsa_circ_0000313, miR‐1224‐3p, and MKNK2 expression was conducted via quantitative real‐time PCR (qRT‐PCR). CCK‐8 assay and flow cytometry were employed to evaluate the proliferative capacity and apoptotic rate of Jurkat cells. ELISA detected inflammatory cytokine secretion. Potential interactions involving miR‐1224‐3p with either hsa_circ_0000313 or MKNK2 were predicted using bioinformatics tools and subsequently validated through dual‐luciferase reporter assays. FISH assay was used to detect subcellular colocalization of hsa_circ_0000313 and miR‐1224‐3p.

**Results:**

We observed that compared to healthy controls, MG patients exhibited increased expression of hsa_circ_0000313 and MKNK2, along with decreased expression of miR‐1224‐3p. Knockdown of hsa_circ_0000313 and MKNK2, along with overexpression of miR‐1224‐3p, inhibited the proliferation and secretion of inflammatory factors in Jurkat cells and promoted their apoptosis. Additionally, miR‐144‐3p was identified as the target miRNA of hsa_circ_0000313, and MKNK2 was identified as the target gene of miR‐1244‐3p. Hsa_circ_AA0000313 was confirmed to be circular and resistant to RNase R digestion. There was significant colocalization of hsa_circ_0000313 with miR‐1224‐3p within the cytoplasm. Inhibition of miR‐1244‐3p reversed the effects of hsa_circ_0000313 knockdown and MKNK2 knockdown on Jurkat cells proliferation, apoptosis, and inflammatory cytokine secretion. In LPS–stimulated Jurkat cells, hsa_circ_0000313 knockdown suppressed the p38 MAPK pathway via the miR‐1224‐3p/MKNK2 axis, which reduced inflammatory cytokine secretion and cell proliferation as well as promoted apoptosis.

**Conclusion:**

Hsa_circ_0000313 is involved in MG progression by regulating the miR‐1224‐3p/MKNK2 axis and can act on the p38MAPK pathway to participate in the progression of MG concomitant inflammatory infections, which may provide a promising therapeutic target in MG.

## 1. Introduction

Myasthenia gravis (MG) is an autoimmune disorder characterized by impaired signal transmission at the neuromuscular junction caused by antibodies to the acetylcholine receptor (AChR), muscle‐specific kinase (MuSK), or other AChR‐associated proteins expressed in postsynaptic membranes, resulting in impaired muscle contraction and clinical manifestations such as ptosis, diplopia, dysphagia, and generalized weakness [1]. Although relevant studies have shown that MG is an antibody‐mediated autoimmune disease, autoantibodies are not detected in about 10% of patients, and the disease is known as seronegative MG (SNMG) [2]. The clinical classification is based on the range of MG symptoms from Type I to Type V, ranging from the mildest ocular muscle weakness to eventual respiratory muscle involvement. Bacterial infections causing respiratory tract infections are a common cause of MG exacerbation [3]. Clinically, acetylcholinesterase inhibitors and combined immunosuppressive therapy are frequently employed; however, long‐term use of this therapeutic strategy may also trigger a series of opportunistic infections [4, 5]. In most clinical cases, the respiratory muscles are not affected. However, weakness in these muscles predisposes patients to respiratory infections [3]. Severe dyspnea can precipitate a life‐threatening myasthenic crisis [6, 7], which is the most critical complication of the disease. Early intervention through prompt diagnosis and management of MG is critical for mitigating disease progression, while increased attention to and further investigation of infectious complications in affected individuals remains imperative.

T cell‐dependent orchestration and B cell‐mediated humoral immunity are fundamental to the pathogenesis of MG, defining it as a classic autoimmune disorder [1]. It was found that MG patients have increased B cell differentiation, an imbalance in CD4^+^ T cell subsets and a highly activated monocyte inflammatory pathway [8]. CD4^+^CD25^+^FoxP3^+^ regulatory T (Treg) cell subsets have been shown to be an important component of the immune system [9]. CD4^+^ T cells are important regulators of acquired immunity, and CD4^+^ T cells can be functionally divided into four subpopulations: T helper 1 cells (Th1), Th2, Th17, and Treg [10]. The pathogenesis of MG involves excessive activation of CD4^+^ T cells and their cytokines [11], which drive antigen‐sensitized B cells to produce autoantibodies [12, 13].

The molecular mechanisms of MG pathogenesis are highly complex and not yet fully elucidated, but in recent years screening for biomarkers could lead to precise treatment of MG patients in the future [14]. Noncoding RNAs (ncRNAs), a diverse category of transcriptional products including long ncRNAs (lncRNAs), circular RNAs (circRNAs), heterogeneous nuclear RNAs (hnRNAs), PIWI–interacting RNAs (piRNAs), ribosomal RNAs (rRNAs), small nuclear RNAs (snRNAs), small nucleolar RNAs (snoRNAs), and transfer RNAs (tRNAs), serve as critical regulators in the molecular mechanisms underlying a spectrum of pathological conditions, notably neurological diseases, malignancies, and cardiovascular disorders [15, 16]. CircRNAs, which are widely expressed in the human genome [17], are covalently closed circular ncRNAs formed through back‐splicing of precursor mRNA, with their circular structure relying on the reverse ligation between the downstream 5′ splice site and the upstream 3′ splice site in intronic regions. Due to the absence of a 3′‐poly‐A tail and a 5′‐cap structure, circRNAs effectively resist degradation by exonucleases such as RNase R, thereby exhibiting higher stability than linear RNAs [17–19]. Research indicates that circRNAs are heterogeneously distributed within cells, exhibiting specific subcellular localization patterns that closely correlate with their biological functions [20]. In the nucleus, circRNAs consist mainly of intronic circRNAs (ciRNAs) and exon‐intron circRNAs (EIciRNAs) [21], which typically regulate gene transcription positively or negatively by interacting with the promoter regions of their parental genes [22]. In the cytoplasm, circRNAs are predominantly composed of exonic circRNAs (ecircRNAs), whose primary function is to act as “molecular sponges” that sequester microRNA (miRNA) and modulate gene expression networks [23]. Cytoplasmic circRNAs perform critical biological functions by acting as miRNA sponges within the competing endogenous RNA (ceRNA) mechanism to regulate gene expression, participating in transcriptional control, and interacting with RNA–binding proteins (RBPs) [16, 24, 25]. miRNA has also been considered as a biomarker for prognosis of disease activity due to its stability, ease of access through blood analysis and specific changes associated with different diseases [26]. Recent studies have discovered protein‐coding circRNAs [27–29]. Research has found that a novel candidate biomarker, hsa_circ5333_4, can be identified based on its characteristic expression profile in peripheral blood from MG patients [30]. Lai et al. [31] demonstrated that the miR‐133/PAX7 axis mediates the pro‐proliferative effect of circ‐FBL upregulation on myogenic cells in MG. CircRNAs have been identified as novel biomarkers in patients with other autoimmune diseases including systemic lupus erythematosus (SLE) [32], with research demonstrating that hsa_circ_0012919 modulates MDA5 expression in CD4^+^ T cells of SLE patients through miR‐125a‐3p regulation [33]. Based on Kong et al.’s [34] microarray analysis of dysregulated circRNAs in MG patients, we selected hsa_circ_0000313 for our study. The study of the circRNA–miRNA–mRNA regulatory axis and protein interaction patterns offers a new perspective for elucidating the molecular mechanisms of MG initiation and development. In this study, we identified that hsa_circ_0000313 possesses a binding site for miR‐1224‐3p, which may potentially target MKNK2. Our findings were designed to elucidate the mechanistic role of hsa_circ_0000313 in MG. Clarification of the regulatory function of the hsa_circ_0000313/miR‐1224‐3p/MKNK2 axis within the p38 MAPK signaling pathway was expected to facilitate the identification of novel therapeutic targets for MG.

## 2. Methods

### 2.1. Screening of circRNAs

Using our research group’s previous circRNA chip sequencing in MG patients and healthy controls to obtain database results, we screened for differentially expressed circRNAs under the conditions of |log_2_FC| > 0.5 and *p*‐value <0.05.

### 2.2. Clinical Samples

A total of 50 individuals diagnosed with MG were recruited from the Second Affiliated Hospital of Harbin Medical University (36 females and 14 males, mean age: 60.42 ± 12.31 years). Diagnosis was established in accordance with accepted clinical criteria for MG. Exclusion criteria comprised a history of infectious diseases or concurrent active autoimmune disorders. In addition, 50 healthy volunteers, matched by gender and age (30 females and 20 males, mean age: 57 ± 9.5years), were enrolled as controls. All control subjects were confirmed to be free of autoimmune conditions. The study protocol received approval from the Ethics Committee of the Second Affiliated Hospital of Harbin Medical University (YJSKY2024‐010), and written informed consent was acquired from every participant prior to enrollment. Peripheral venous blood (5 mL) was collected from each subject using EDTA–containing vacuum tubes.

### 2.3. RNA Extraction, Reverse Transcription, and Quantitative Real‐Time PCR (qRT‐PCR)

Peripheral blood mononuclear cells (PBMCs) were isolated from whole blood using lymphocyte separation medium (TBD, Tianjin, China) according to the manufacturer’s instructions. Total RNA was isolated from PBMCs and Jurkat cells using Trizol reagent (SEVEN, Beijing, China) and quantified with a NanoDrop ND‐1000 system (Thermo Fisher Scientific, Waltham, USA). Subsequently, 1 μg RNA was reverse transcribed into complementary DNA (cDNA) using a reverse transcription kit (SEVEN, Beijing, China), with random primers employed for circRNA and mRNA and stem‐loop primers for miRNA. The expression levels of circRNAs, miRNAs, and mRNAs were quantified by qRT‐PCR using SYBR Green qPCR MasterMix (SEVEN, Beijing, China), with GAPDH and U6 serving as endogenous controls. Each 20 μL reaction contained 1 μL of cDNA, 10 μL of 2 × SYBR Green qPCR Mix, 0.4 μL each of forward and reverse primers, and 8.2 μL of RNase‐free H_2_O. The thermal cycling conditions were: initial denaturation at 95°C for 30s, followed by 40 cycles of 95°C for 20 s and 60°C for 20 s. Relative gene expression was calculated using the 2^−*ΔΔ*Ct^ method. The primer sequences for qRT‐PCR are listed in Table 1.

**Table 1:** Primers used for qRT‐PCR analysis.

Gene	Primers sequences
*hsa_circ_0000313*	Forward (5′–3′): GGAGTCTCAGGAGGAGGCT
	Reverse (5′–3′): AGCGCCGTCCACACAGCA

*miR-1224-3p* (Stem‐loop method)	Reverse transcription primer: GTCGTATCCAGTGCAGGGTCCGAGGTATTCGCACTGGATACGACCTGAGG
	Forward (5′–3′): GCGCCCCACCTCCTCTCT
	Reverse (5′–3′): AGTGCAGGGTCCGAGGTATT

*miR-1200* (Stem‐loop method)	Reverse transcription primer: GTCGTATCCAGTGCAGGGTCCGAGGTATTCGCACTGGATACGACGAGGCT
	Forward (5′–3′): GCGCTCCTGAGCCATTCTG
	Reverse (5′–3′): AGTGCAGGGTCCGAGGTATT

*MKNK2*	Forward (5′–3′): CCAGCCGAACTTCAGGGTTT
	Reverse (5′–3′): CGTCCGGGATGTCAATGGG

*GAPDH*	Forward (5′–3′): GATGTTCGTCATGGGTGTGAA
	Reverse (5′–3′): GGCATGGACTGTGGTCATGAG

*U6*	Forward (5′–3′): CTCGCTTCGGCAGCACA
	Reverse (5′–3′): AACGCTTCACGAATTTGCGT

### 2.4. Cell Culture

Two human cell lines—Jurkat cells (a human CD4^+^ T cell leukemia cell line; RRID: CVCL_0065) and 293T cells (a human embryonic kidney epithelial cell line; RRID: CVCL_0063) were purchased from the BeNa Culture Collection (Henan, China). Jurkat cells were cultured in RPMI 1640 medium (Gibco, USA), while 293T cells were cultured in Dulbecco’s modified eagle’s medium (DMEM; Gibco, USA). Both media were supplemented with 10% fetal bovine serum (FBS; Gibco, USA) and 1% penicillin–streptomycin mixture (SEVEN, Beijing, China). All cell cultures were maintained at 37°C in a humidified atmosphere containing 5% CO_2_. Culture media were refreshed every 1–2 days based on the cells’ growth characteristics, and cells in the logarithmic growth phase were collected for subsequent experiments. Both cell lines were confirmed to be free of mycoplasma contamination.

### 2.5. Cell Transfection and LPS Treatement

Small interfering RNAs (siRNAs), their negative controls (siRNA‐NC), miRNA mimics, corresponding negative controls, and miRNA inhibitors with their negative controls were designed as well as manufactured by Hanbio Biotechnology (Shanghai, China) and GenePharma (Shanghai, China). The sequences are listed in Table 2. Jurkat cells were seeded into 6‐well plates and cultured for 24 h before transfection. When Jurkat cells confluence reached 60%–80%, transfection was performed using siRNA‐mate plus transfection reagent (GenePharma, Shanghai, China) according to the manufacturer’s instructions to introduce siRNA, miRNA mimics, and miRNA inhibitors into the Jurkat cells. The transfection mixture consisted of 8.5 μL of buffer, 75 pmol of siRNA/miRNA, and 7.5 μL of the siRNA‐mate plus transfection reagent. After 6 h of transfection, the supernatant of transfected Jurkat cells was replaced with fresh complete medium. Subsequently, qRT‐PCR analysis was applied to evaluate transfection efficiency, with the maximum transfection efficiency detected at 48 h after transfection. For LPS treatment, Jurkat cells were treated with 10 μg/mL LPS (Solarbio, Beijing, China) and cultured for 48 h.

**Table 2:** Sequences of siRNA, miRNA mimics, and miRNA inhibitors.

Gene	Sequences (5′–3′)
*si-circ_0000313-1#*	Sense: GGCUGGAGGUCUCGUCCCATT
	Antisense: UGGGACGAGACCUCCAGCCTT

*si-circ_0000313-2#*	Sense: CUGGAGGUCUCGUCCCAGCTT
	Antisense: GCUGGGACGAGACCUCCAGTT

*si-circ_0000313-3#*	Sense: GGAGGUCUCGUCCCAGCCCUC
	Antisense: GAGGGCUGGGACGAGACCUCC

*si-MKNK2-1#*	Sense: GCCGUCAAGAUCAUUGAGATT
	Antisense: UCUCAAUGAUCUUGACGGCTT

*si-MKNK2-2#*	Sense: CCUUGGACUUUCUGCAUAATT
	Antisense: UUAUGCAGAAAGUCCAAGGTT

*si-MKNK2-3#*	Sense: CCUGCCAGAACAUGCUGUUTT
	Antisense: AACAGCAUGUUCUGGCAGGTT

*Si-NC*	Sense: UUCUCCGAACGUGUCACGUTT
	Antisense: ACGUGACACGUUCGGAGAATT

*miR-1224-3p-mimics*	Sense: CCCCACCUCCUCUCUCCUCAG
	Antisense: CUGAGGAGAGAGGAGGUGGGG

*miR-1224-3p inhibitor*	Antisense: CUGAGGAGAGAGGAGGUGGGG

*miR-NC*	Sense: UCACAACCUCCUAGAAAGAGUAGA
	Antisense: UCUACUCUUUCUAGGAGGUUGUGA

### 2.6. Dual‐Luciferase Reporter Assay

Binding sites of miR‐1224‐3p that target hsa_circ_0000313 were predicted via the CircInteractome database. Binding sites of miR‐1224‐3p that target MKNK2 were predicted via the TargetScanHuman 8.0 database. The wild‐type (WT) luciferase reporter vectors for hsa_circ_0000313 and MKNK2 (designated as hsa_circ_0000313‐WT and MKNK2‐3′UTR‐WT, respectively) were constructed by inserting the corresponding sequences of hsa_circ_0000313 and MKNK2 containing the miR‐1224‐3p binding sites into the pmirGLO luciferase reporter vector (GenePharma, Shanghai, China). Concurrently, mutant (MUT) luciferase reporter vectors for hsa_circ_0000313 and MKNK2 (designated hsa_circ_0000313‐MUT and MKNK2‐3′UTR‐MUT, respectively) were produced by mutating the miR‐1224‐3p binding sites. Subsequently, 293T cells were cotransfected with each constructed reporter plasmid together with either miR‐1224‐3p mimics or miR‐NC, as detailed in Supporting Information 1: Tables S2 and S3.

Luciferase activity was measured using a Dual‐Luciferase Reporter Assay Kit II (Beyotime, Shanghai, China). Signals from firefly and Renilla luciferase were sequentially quantified in cell lysates with an Infinite M200 (Switzerland, Tecan) and recorded as relative light units (RLUs). First, the firefly luciferase activity value of each sample well was divided by its corresponding Renilla luciferase activity value to obtain the relative luciferase activity, which normalized for variations in transfection efficiency. Then, the average relative activity of each experimental group was divided by the average relative activity of the control group for normalization.

### 2.7. RNase R Linear RNA Digestion Experiment

Total RNA was extracted from Jurkat cells using Trizol reagent and diluted to a final concentration of 500 ng/μL. Using the RNase R kit (Cat. Number R0301; GENESEED, Guangzhou, China). Reaction mixtures were prepared according to the treatment groups: for the RNase R group, 4 μL RNA was mixed with 0.3 μL RNase R, 2 μL 10 × reaction buffer, and 13.7 μL RNase‐free water; for the Mock group, 4 μL RNA was mixed with 2 μL 10 × reaction buffer and 14 μL RNase‐free water. After incubation at 37°C for 10 min, qRT‐PCR was performed to measure the expression levels of hsa_circ_0000313 and FADS2 in both groups.

### 2.8. Nucleoplasmic Separation Experiment

Cytoplasmic and nuclear RNA was extracted from Jurkat cells using a cytoplasmic‐nuclear RNA purification kit (Beyotime, Shanghai, China). The reaction system was as follows: to 20 μL of cell pellet, 200 μL of Solution A, and 5 μL of RNase inhibitor were added and vortexed until fully resuspended. Subsequently, 10 μL of Solution B was added, and the mixture was centrifuged. The resulting supernatant contained the cytoplasmic fraction, while the pellet contained the nuclear fraction. RNA was then extracted from both fractions using the Trizol method. The expression of hsa_circ_0000313 in the nuclear and cytoplasmic fractions was analyzed by qRT‐PCR, using GAPDH and U6 as the internal controls for the cytoplasmic and nuclear fractions, respectively.

### 2.9. Flow Cytometry

Following the manufacturer’s protocol, cell apoptosis was evaluated via FITC Annexin V Apoptosis Detection Kit I (Becton‐Dickinson, FL, NJ, USA). Cells were washed twice with ice‐cold 1 × PBS, then resuspended in binding buffer at a density of 1 × 10^6^ cells/mL. Thereafter, 5 μL of Annexin V‐FITC and 5 μL of propidium iodide (PI) were added to 100 μL of the aforementioned cell suspension. The mixture was incubated for 15 min at room temperature under dark conditions, and the apoptosis rate was subsequently analyzed using a flow cytometer (Cytek Biosciences, USA).

### 2.10. CCK‐8 Assay

Cell proliferation was evaluated using a CCK‐8 kit (SEVEN, Beijing, China). Transfected cells were seeded in 96‐well plates; 10 μL CCK‐8 reagent per well was added at 24, 48, 72, and 96 h posttransfection, followed by incubation at 37°C for 1 h, and the optical density (OD) of each well was measured at a wavelength of 450 nm.

### 2.11. ELISA

After the respective treatments, cells culture supernatants were collected, and the levels of inflammatory cytokine (TNF‐α, IL‐6, and IL‐10) were quantified with an ELISA kit (JONLNBIO, Shanghai, China) by measuring the absorbance at 450 nm according to the manufacturer’s instructions. A standard curve (standard concentration vs. OD) was generated to calculate the concentrations of the samples.

### 2.12. Western Blot Assay

Total protein was extracted using RIPA lysis buffer (Beyotime, Shanghai, China). The protein concentration was determined with a BCA assay kit (Beyotime, Shanghai, China). Then, 5 × loading buffer (Epizyme, Shanghai, China) was added to the lysate, followed by boiling at 100°C for 10 min. Subsequently, proteins were separated by 10% SDS‐PAGE gels (Servicebio, Wuhan, China) and then were transferred onto PVDF membranes. Following blocking with Rapid Block Buffer (Epizyme, Shanghai, China) for 30 min at room temperature, the membranes were incubated overnight at 4°C with the following primary antibodies: rabbit anti‐MKNK2 (1:1000 dilution; Proteintech), rabbit anti‐phosphorylated (p)‐p38 (1:1000 dilution; Proteintech), rabbit anti‐p38 (1:2000 dilution; Proteintech), rabbit anti‐PCNA (1:5000 dilution; Proteintech), rabbit anti‐BCL‐2 (1:2000 dilution; Proteintech), rabbit anti‐Bax (1:2000 dilution; Proteintech), rabbit anti‐β‐actin (1:5000 dilution; Proteintech), and rabbit anti‐GAPDH (1:5000 dilution; Proteintech). Thereafter, the membranes were incubated with horseradish peroxidase (HRP)–conjugated secondary antibodies for 1 h at RT. After washing with TBST, protein bands were visualized using an enhanced chemiluminescence (ECL) reagent (Wanleibio, Beijing, China) and were quantified by densitometric analysis using ImageJ software (National Institutes of Health, USA).

### 2.13. FISH Assay

The FISH technique was used to determine the localization of hsa_circ_0000313 with miR‐1224‐3p in the cells. Hsa_circ_0000313 was labeled by a cy3 probe and the sequence was: CCCCACCTCCTCTCTCCTCAG. MiR‐1224‐3p was labeled by FAM probe and the sequence was: GCTGGGACGAGACCTCCAGCCC. The probes were designed and synthesized by GENESEED and a FISH kit (GENESEED, Guangzhou, China) was utilized to detect the signal of the probes according to the manufacturer’s instructions. Cells grown on glass coverslips were fixed with 4% paraformaldehyde and permeabilized using 0.5% Triton X‐100. After prehybridization, samples were incubated overnight at 37°C in a hybridization buffer containing both the cy3‐labeled hsa_circ_0000313 probe and the FAM‐labeled miR‐1224‐3p probe (each at a concentration of 50 nM) for dual‐color FISH colocalization analysis. Following hybridization and washes, cell nuclei were counterstained with 4′,6‐diamidino‐2‐phenylindole (DAPI) for 5 min at room temperature. Cell nuclei were counterstained with DAPI. Images were taken with a laser scanning confocal microscope LSM800 (Carl Zeiss, Germany). The cell experiments were independently repeated three times.

### 2.14. circRNA Validation Assay

Genomic DNA (gDNA) was isolated from PBMCs using a gDNA extraction kit (Servicebio, Wuhan, China) according to the manufacturer’s instructions. Using cDNA and gDNA as templates, quantitative PCR (qPCR) was performed with divergent and convergent primers specifically designed to span the backsplice junction of the circRNA (Sangon Biotech, Shanghai, China), sequences listed in Table 3. Amplification products were analyzed on an agarose gel, with sequence verification by Sanger sequencing.

**Table 3:** Divergent and convergent primer sequences.

Gene	Primers sequences
*hsa_circ_0000313-circ-hF*	Forward (5′–3′): ACCAGGGTGGAGCTGAGAAGA
*hsa_circ_0000313-circ-hR*	Reverse (5′–3′): CACAGTAAGGGCAGGTTCAGACA
*hsa_circ_0000313-Linear-hF*	Forward (5′–3′): TCTCCTGTCTGAACCTGCCCTTAC
*hsa_circ_0000313-Linear-hR*	Reverse (5′–3′): CAGCTCCACCCTGGTCCTCA
*GAPDH-circ-hF*	Forward (5′–3′): ACACCCACTCCTCCACCTTT
*GAPDH-circ-hR*	Reverse (5′–3′): AATCCGTTGACTCCGACCTT
*GAPDH-hF*	Forward (5′–3′): TGACAACTTTGGTATCGTGGAAGG
*GAPDH-hR*	Reverse (5′–3′): AGGCAGGGATGATGTTCTGGAGAG

## 3. Statistical Analysis

All data were analyzed and graphed using GraphPad Prism software, and were expressed as mean ± standard deviation (SD). Differences between two groups were evaluated using Student’s *t*‐test (for normally distributed data) or the Mann–Whitney *U* test (for nonnormally distributed data). Comparisons among three or more groups were conducted via one‐way analysis of variance (ANOVA) and followed by Tukey’s post hoc test for multiple comparisons. Correlations between the expression levels of circ_0000313, miR‐1224‐3p, and MKNK2 were analyzed using Pearson correlation analysis. Statistical significance was defined as *p* < 0.05.

## 4. Results

### 4.1. hsa_circ_0000313 Exhibits High Expression in Patients With MG

A total of 28 differentially expressed circRNAs were identified from the dataset, of which eight were upregulated and 20 were downregulated. The results are shown in Supporting Information 1: Table S1. hsa_circRNA_0000313 was selected as the target gene for subsequent research. Further database queries revealed that its circBase ID is hsa_circ_0000313. It is located on chromosome 11 (chr11), originates from the parental gene FADS2, has a genomic length of 318 nt and is classified as an exon–exon circRNA.

Sanger sequencing confirmed the back‐splice junction of hsa_circ_0000313 and the sequence is consistent with circBase database annotation (http://www.circbase.org/; Figure 1A). Electrophoretic analysis showed that the expected band was amplified by the divergent primers only from the cDNA template, with no amplification observed from gDNA (Figure 1B). RNase R digestion assays also confirmed the RNA’s circular nature, demonstrating its resistance to RNase R. When the divergent primers were used, a band of the correct size was detected in untreated cDNA and its signal remained stable after RNase R treatment, while no amplification product was obtained from the gDNA template. In contrast, when the convergent primers were used, the major band amplified from untreated cDNA exhibited a higher molecular weight than expected, consistent with pre‐mRNA amplification, and this band was completely eliminated following RNase R treatment (Figure 1C). To further validate these findings, total RNA extracted from Jurkat cells was digested with RNase R, and the expression levels of hsa_circ_0000313 and its linear parental gene FADSD2 mRNA were subsequently measured by qRT‐PCR. Total RNA isolated from cells was treated with RNase R, using linear FADS2 mRNA as a control. The results indicated that the expression level of hsa_circ_0000313 remained stable after RNase R treatment, while linear FADS2 expression was significantly decreased, confirming that it is produced through selective splicing of FADS2 transcripts (Figure 1D). The nucleoplasmic separation experiment revealed that hsa_circ_0000313 is predominantly localized in the cytoplasm (Figure 1E). Furthermore, qRT‐PCR analysis of RNA isolated from the blood of 50 MG patients and 50 healthy controls demonstrated that hsa_circ_0000313 expression was statistically significantly higher in the MG group compared with healthy controls group (Figure 1F).

Figure 1hsa_circ_0000313 exhibits high expression in patients with MG. (A) Sanger sequencing validation of the hsa_circ_0000313 splicing junction. (B) Amplification of hsa_circ_0000313 and GAPDH using convergent and divergent primers with cDNA and gDNA from Jurkat cells as templates. (C) Amplification of hsa_circ_0000313 using convergent and divergent primers with RNase R‐digested (+) or undigested (−) Jurkat cell cDNA, as well as gDNA, as templates. (D) Changes in the expression levels of hsa_circ_0000313 and its parental gene before and after RNase R treatment. (E) Subcellular localization of hsa_circ_0000313 in Jurkat cells determined by qRT‐PCR. (F) Expression levels of hsa_circ_0000313 in peripheral blood from 50 MG patients and 50 healthy controls detected by qRT‐PCR. (G) Cell proliferation was assessed by CCK‐8 assay. The protein level of PCNA was determined by western blot. (H) Analysis of the apoptotic cell rate by flow cytometry. (I) The protein levels of Bcl‐2 and Bax were determined by western blot. (J) ELISA for quantifying inflammatory cytokines.  ^∗^ *p* < 0.05,  ^∗∗^ *p* < 0.01,  ^∗∗∗^ *p* < 0.001, and  ^∗∗∗∗^ *p* < 0.0001.

**Figure:**
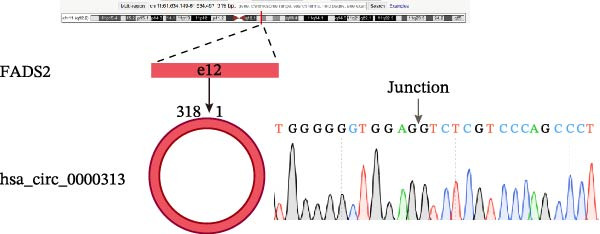
(A)

**Figure:**
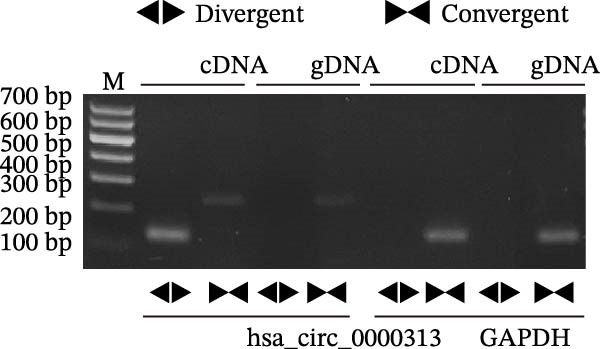
(B)

**Figure:**
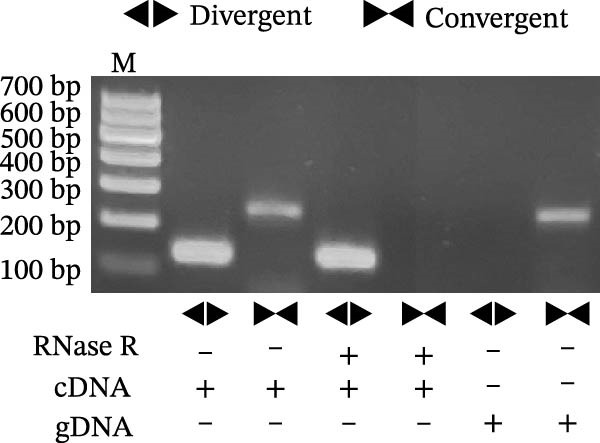
(C)

**Figure:**
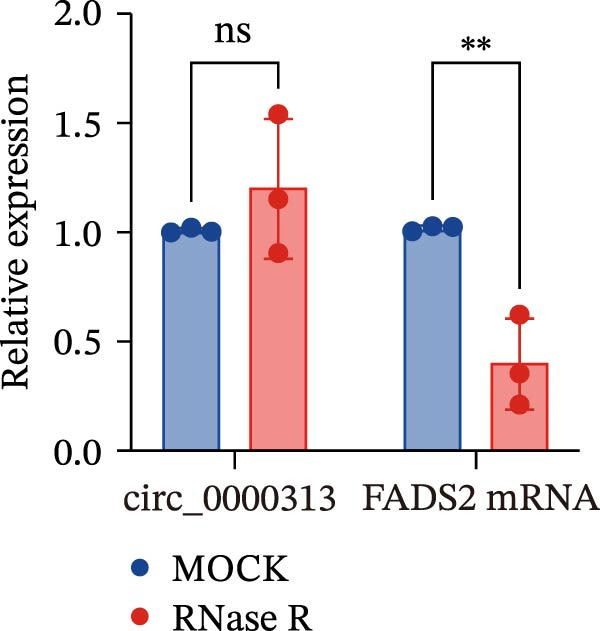
(D)

**Figure:**
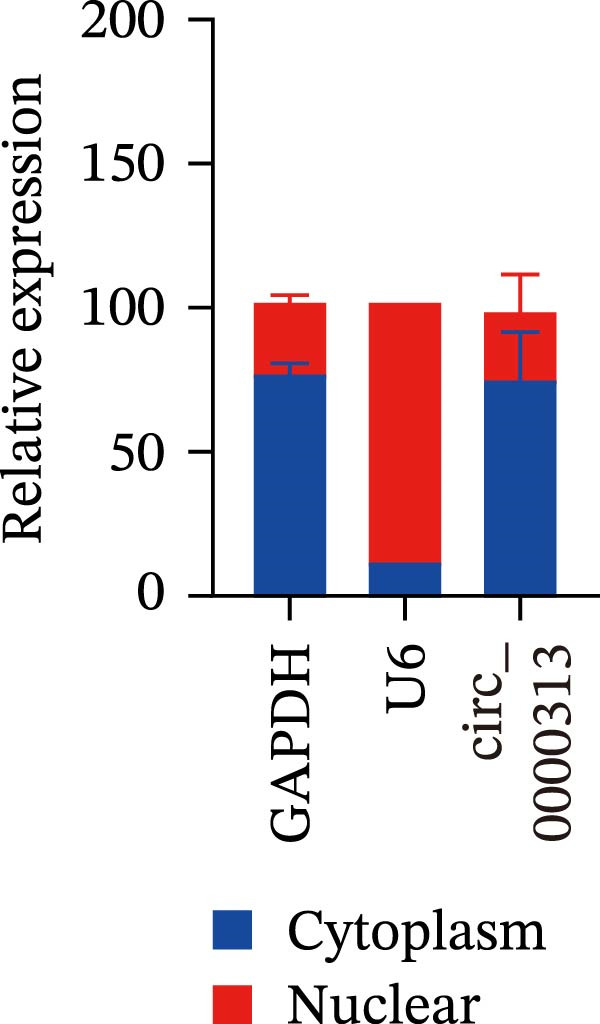
(E)

**Figure:**
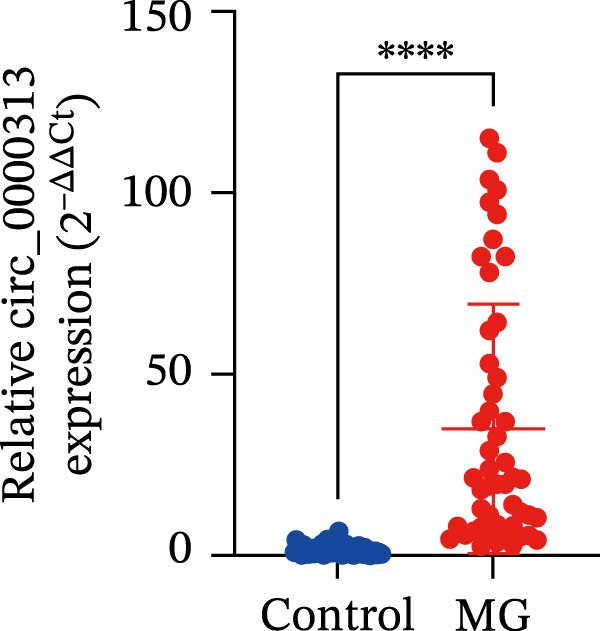
(F)

**Figure:**
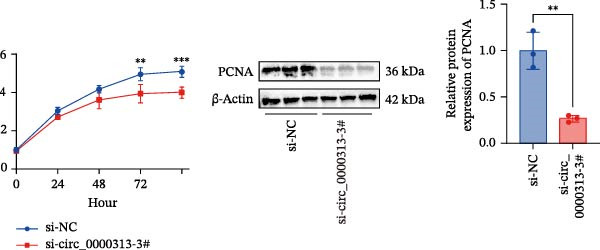
(G)

**Figure:**
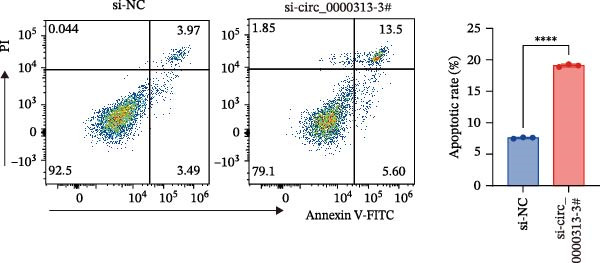
(H)

**Figure:**
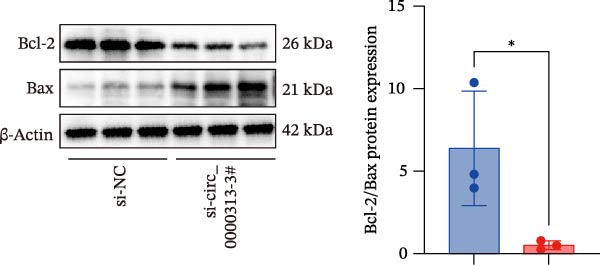
(I)

**Figure:**
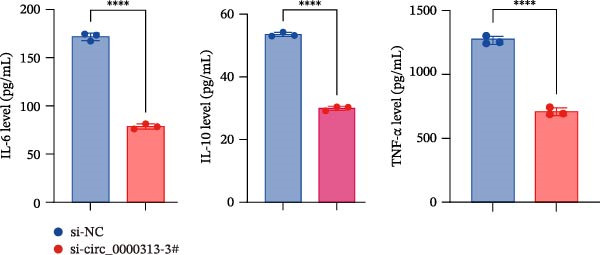
(J)

### 4.2. Knockdown of hsa_circ_0000313 Inhibited Cell Proliferation, Suppressed Inflammatory Cytokine Secretion, and Promoted Cell Apoptosis

To investigate the biological function of hsa_circ_0000313 in MG, knockdown was performed in Jurkat cells using three siRNAs. si‐circ_0000313‐3# showed the strongest inhibitory effect (Supporting Information 2: Figure S1A). Its transfection significantly suppressed cell proliferation at 48, 72, and 96 h and reduced proliferating cell nuclear antigen (PCNA) expression (Figure 1G). Flow cytometry revealed increased apoptosis (Figure 1H), accompanied by decreased levels of the antiapoptotic protein Bcl‐2 and increased levels of the pro‐apoptotic molecule BAX, decreasing the Bcl‐2/BAX ratio (Figure 1I). ELISA indicated significantly reduced secretion of TNF‐α, IL‐10, and IL‐6 (Figure 1J).

### 4.3. hsa_circ_0000313 was a Sponge of miR‐1224‐3p

The biological functions of circRNAs are closely associated with their subcellular localization. Previous studies have shown that the vast majority of circRNAs are located in the cytoplasm and regulate gene expression through the ceRNA mechanism by acting as miRNA sponges. In this study, the nucleoplasmic separation experiment confirmed that hsa_circ_0000313 is predominantly enriched in the cytoplasm. Based on this finding, we further investigated whether hsa_circ_0000313 can exert its corresponding biological functions by sponging miRNAs.

Potential target miRNAs of hsa_circ_0000313 were predicted using two databases, CircInteractome (https://circinteractome.nia.nih.gov) and circBank (http://www.circbank.cn/index.html). The intersection of the predicted target miRNAs from these two databases identified two common miRNAs: miR‐1224‐3p and miR‐1220 (Figure 2A). Analysis of peripheral blood RNA by qRT‐PCR in 50 patients with MG and 50 healthy controls revealed that expression of both miR‐1224‐3p and miR‐1220 was decreased in the MG, with a particularly pronounced reduction in miR‐1224‐3p (Figure 2B). Pearson correlation analysis showed that the expression of hsa_circ_0000313 was negatively correlated with the expression of miR‐1224‐3p (Figure 2C). Therefore, we selected miR‐1224‐3p for further study. Complementary binding sites for MiR‐1224‐3p and hsa_circ_0000313 are shown in Figure 2A. Transfection with si‐hsa_circ_0000313‐3# increased the expression of miR‐1224‐3p (Figure 2D). Transfection with miR‐1224‐3p‐mimic and miR‐1224‐3p‐inhibitor increased and decreased miR‐1224‐3p expression, respectively (Figure 2E).The reduced expression of miR‐1224‐3p in the si‐circ_0000313‐3# + miR‐1224‐3p‐inhibitor group demonstrated that the miR‐1224‐3p‐inhibitor reversed the upregulatory effect of si‐circ_0000313‐3# on miR‐1224‐3p (Figure 2F). The dual‐luciferase reporter assay showed that transfection with the miR‐1224‐3p mimic inhibited the luciferase activity of circ_0000313‐WT, but had no notable effect on that of circ_0000313‐MUT (Figure 2G). Subsequent FISH assay confirmed the significant colocalization of hsa_circ_0000313 and miR‐1224‐3p in the cytoplasm of Jurkat cells, which is consistent with our previous data (Figure 3A). Quantitative analysis using the Coloc 2 plugin showed that in the pixel intensity scatter plot of the two RNA signals, the data points exhibited a comet‐shaped distribution extending from the lower left to the upper right, indicating a high spatial correlation between the hsa_circ_0000313‐cy3 signal and the miR‐1224‐3p‐FAM signal (Figure 3B). Analysis of the cytoplasmic RNA signals yielded a Pearson’s correlation coefficient of 0.8744298 ± 0.004972678. Manders’ overlap coefficients indicated that approximately 88.2% of the hsa_circ_0000313‐cy3 signal colocalized with the miR‐1224‐3p‐FAM signal, with Manders’ colocalization coefficient = 0.882693 ± 0.007698 (Figure 3C). The intensity profiles of the two RNA signals exhibited highly synchronized fluctuation patterns where the peaks of hsa_circ_0000313‐cy3 corresponded to those of miR‐1224‐3p‐FAM, and their signal intensities showed overlap across most regions (Figure 3D).

Figure 2hsa_circ_0000313 was a sponge of miR‐1224‐3p. (A) CircInteractome and circBank predicted two potential target miRNAs of hsa_circ_0000313 and provided the binding sequences of miR‐1224‐3p and miR‐1200 with hsa_circ_0000313. (B) Expression levels of miR‐1224‐3p and miR‐1200 in peripheral blood from 50 MG patients and 50 healthy controls detected by qRT‐PCR. (C) Correlation between the expression of hsa_circ_0000313 and miR‐1224‐3p was assessed in peripheral blood from 50 MG patients by Pearson’s correlation test. (D–F) The effect of si‐circ_0000313‐3#, miR‐144‐3p mimic, and miR‐144‐3p inhibitor on miR‐144‐3p expression was analyzed by qRT‐PCR. (G) Dual‐luciferase reporter assay was performed to identify the association of hsa_circ_0000313 with miR‐1224‐3p.  ^∗^ *p* < 0.05,  ^∗∗^ *p* < 0.01,  ^∗∗∗^ *p* < 0.001, and  ^∗∗∗∗^ *p* < 0.0001.

**Figure:**
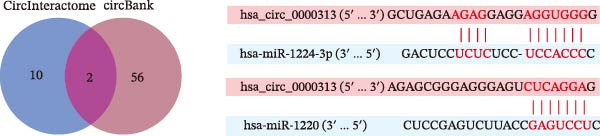
(A)

**Figure:**
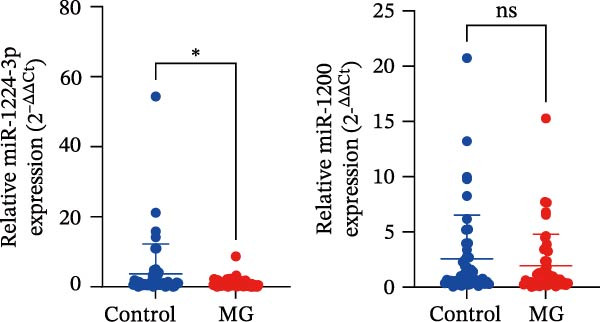
(B)

**Figure:**
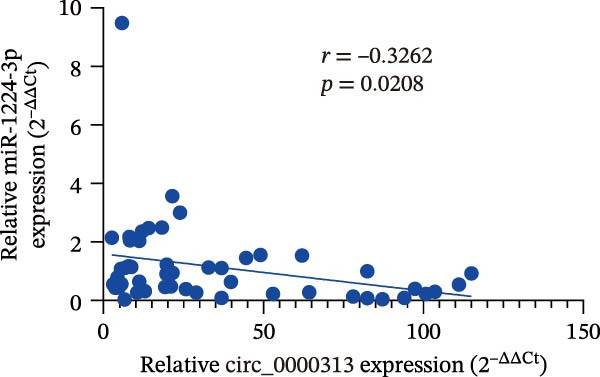
(C)

**Figure:**
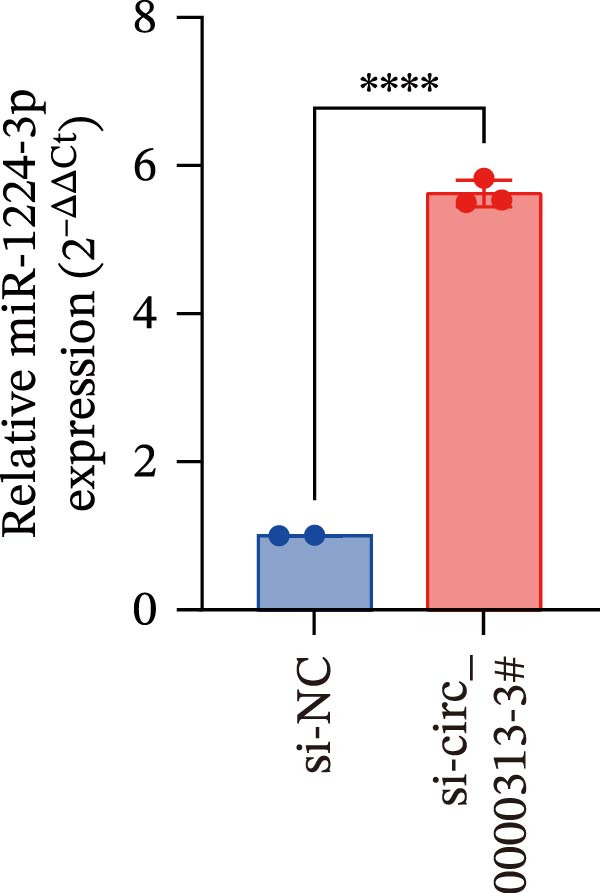
(D)

**Figure:**
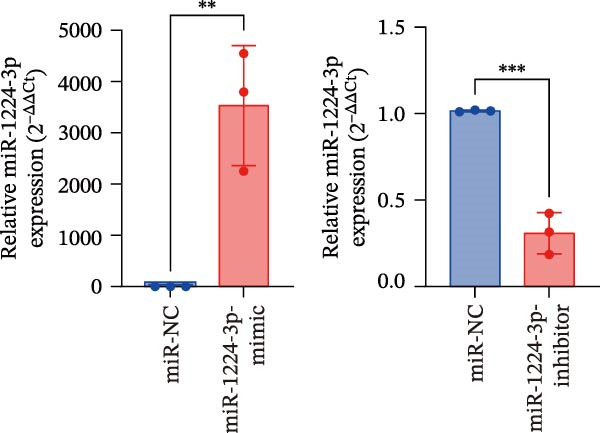
(E)

**Figure:**
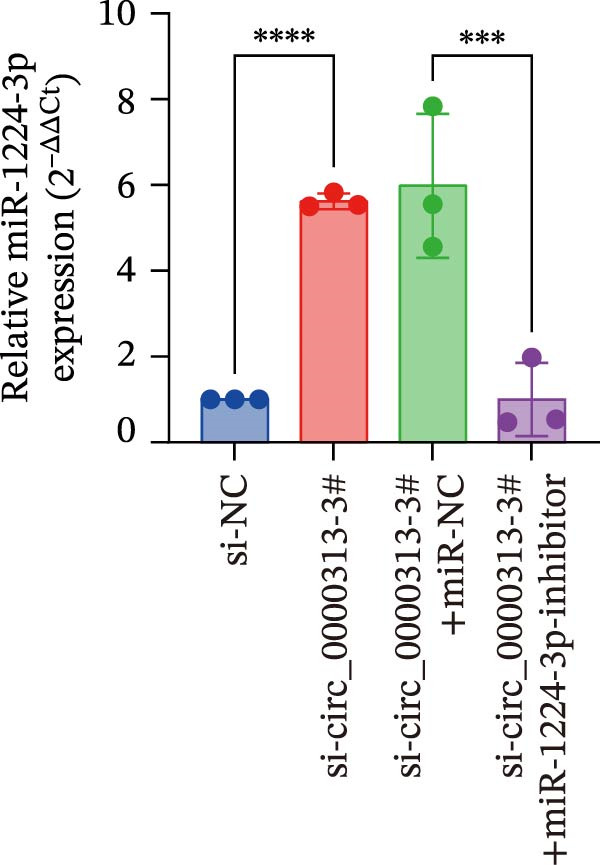
(F)

**Figure:**
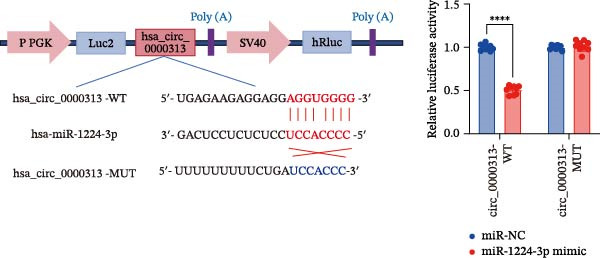
(G)

Figure 3hsa_circ_0000313 co‐localizes with miR‐1224‐3p in the cytoplasm. (A) Jurkat cell FISH detection, red: hsa_circ_0000313‐cy3; green: miR‐1224‐3p‐FAM; blue: DAPI staining for nuclear localization. (B) Pixel intensity scatter plot. (C) Violin plot of Pearson correlation coefficient and Manders overlap coefficient. (D) Peak line graph of signal strength.

**Figure:**
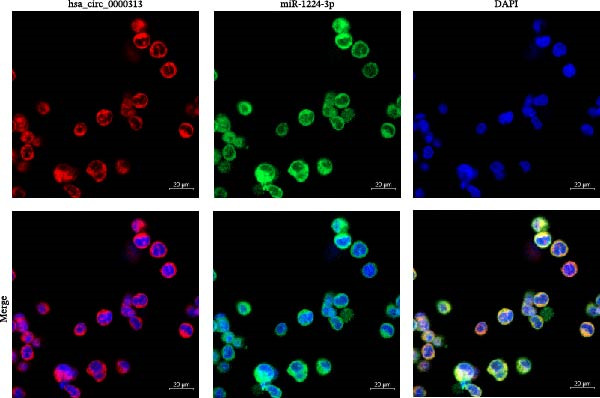
(A)

**Figure:**
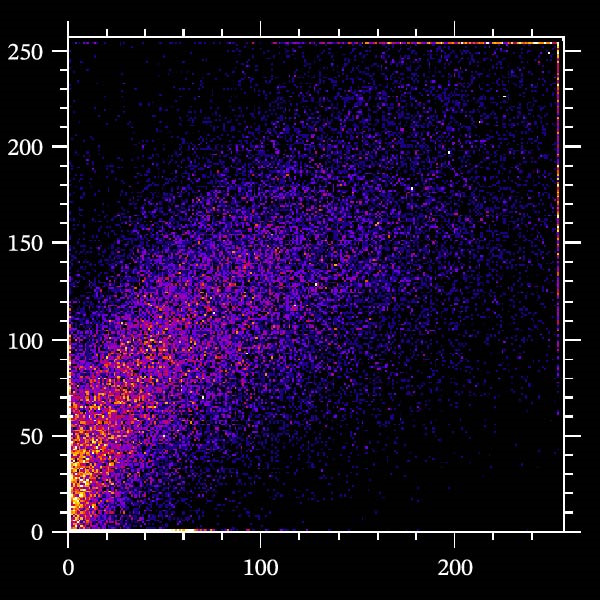
(B)

**Figure:**
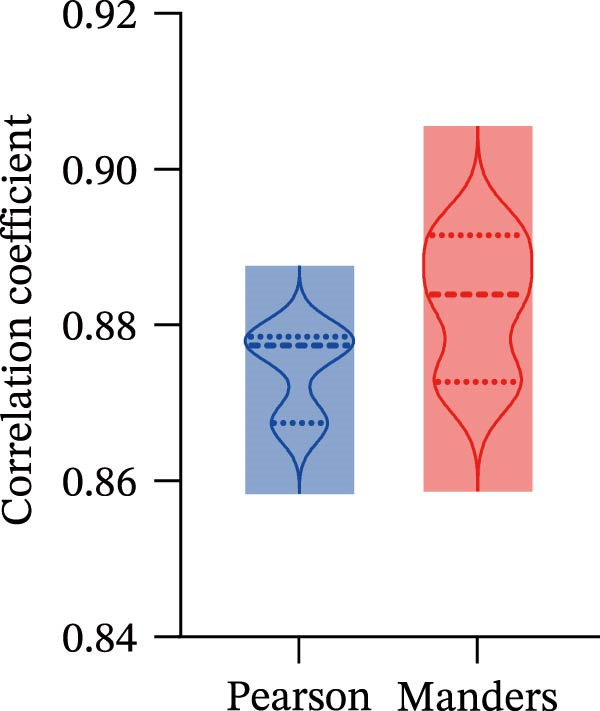
(C)

**Figure:**
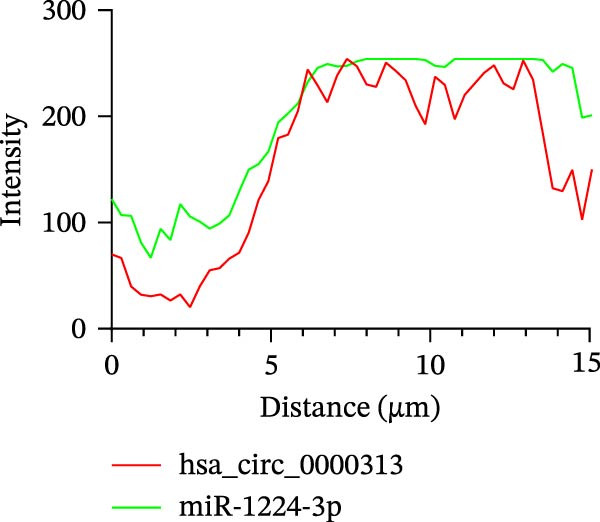
(D)

Collectively, these data indicated that miR‐1224‐3p is a direct target of hsa_circ_0000313.

### 4.4. hsa_circ_0000313 Modulated Cellular Behaviors by Regulating miR‐1224‐3p

Transfection of Jurkat cells with a miR‐1224‐3p mimic significantly inhibited cell proliferation and reduced PCNA expression at 48, 72, and 96 h (Figure 4A). Flow cytometry indicated increased apoptosis (Figure 4C), accompanied by decreased levels of the antiapoptotic protein Bcl‐2 and increased levels of the proapoptotic molecule BAX, decreasing the Bcl‐2/BAX ratio (Figure 4D). ELISA indicated decreased secretion of TNF‐α, IL‐10, and IL‐6(Figure 5A). These results indicated that miR‐1224‐3p and hsa_circ_0000313 exerted opposite effects on Jurkat cell proliferation, inflammatory cytokine secretion, and apoptosis.

Figure 4hsa_circ_0000313 modulated cellular behaviors by regulating miR‐1224‐3p. Jurkat cells were divided into si‐NC group, si‐ circ_0000313‐3# group, miR‐NC group, miR‐1224‐3p mimic group, si‐ circ_0000313‐3# + miR‐NC group, and si‐ circ_0000313‐3# + miR‐1224‐3p inhibitor group. (A, B) Cell proliferation was assessed by CCK‐8 assay. The protein level of PCNA was determined by western blot. (C–F) Analysis of the apoptotic cell rate by flow cytometry. The protein levels of Bcl‐2 and Bax were determined by western blot.  ^∗^ *p* < 0.05,  ^∗∗^ *p* < 0.01,  ^∗∗∗^ *p* < 0.001, and  ^∗∗∗∗^ *p* < 0.0001.

**Figure:**
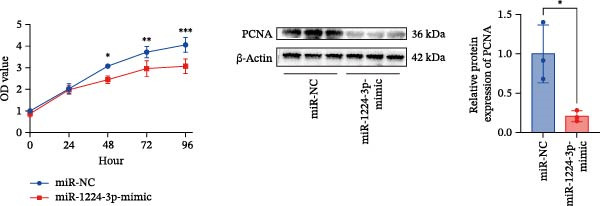
(A)

**Figure:**
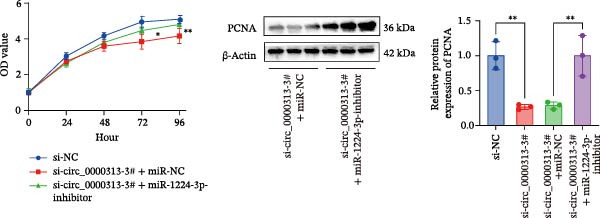
(B)

**Figure:**
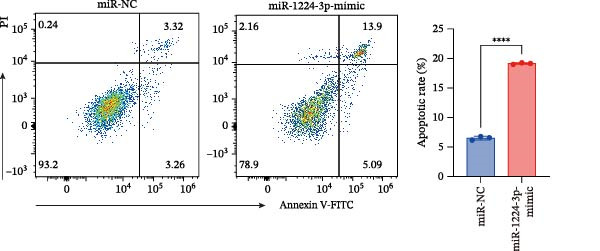
(C)

**Figure:**
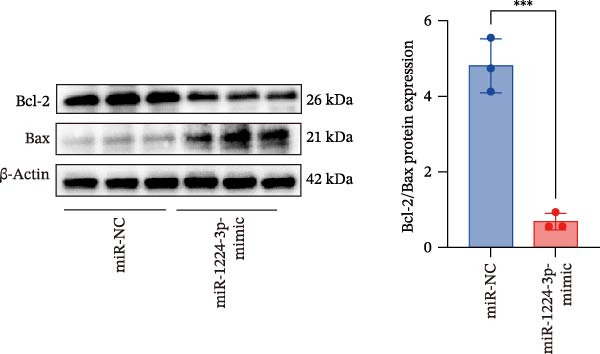
(D)

**Figure:**
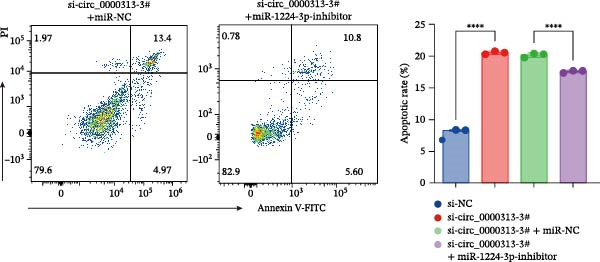
(E)

**Figure:**
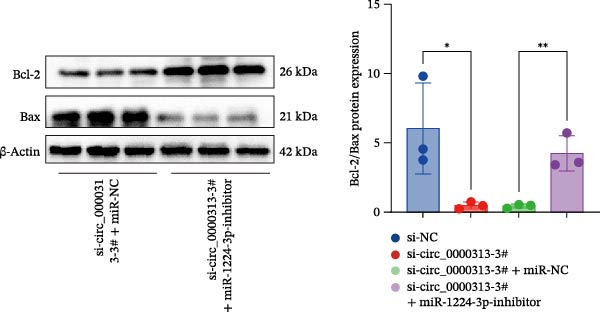
(F)

Figure 5hsa_circ_0000313 modulated cellular behaviors by regulating miR‐1224‐3p. (A, B) ELISA for quantifying inflammatory cytokines.  ^∗∗^ *p* < 0.01,  ^∗∗∗^ *p* < 0.001, and  ^∗∗∗∗^ *p* < 0.0001.

**Figure:**
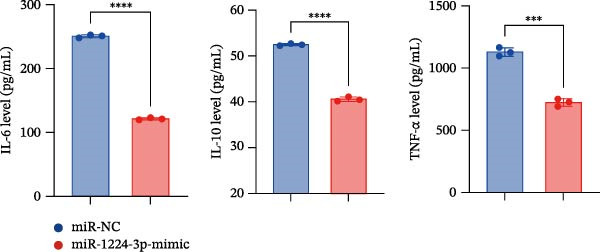
(A)

**Figure:**
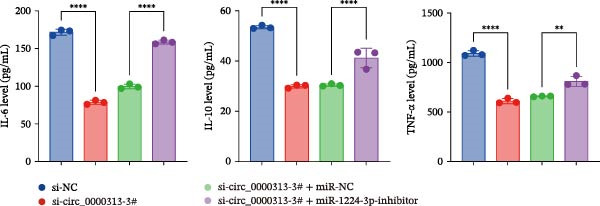
(B)

To determine whether hsa_circ_0000313 regulates cellular behaviors by targeting miR‐1224‐3p, Jurkat cells were transfected with si‐circ_0000313‐3# + miR‐1224‐3p inhibitor. CCK‐8 assays showed enhanced cell proliferation at 72 and 96 h and increased PCNA protein expression (Figure 4B). Flow cytometric analysis revealed a decrease in apoptosis (Figure 4E), accompanied by elevated Bcl‐2 and reduced BAX levels, increasing the Bcl‐2/BAX ratio (Figure 4F). ELISA indicated increased secretion of the inflammatory cytokines TNF‐α, IL‐10, and IL‐6 (Figure 5B). The miR‐1224‐3p inhibitor reversed the suppressive effects of si‐circ_0000313 on cell proliferation, its promoting effect on apoptosis, and its inhibitory action on inflammatory cytokine secretion. Together, these results suggest that hsa_circ_0000313 exerts its functions by targeting miR‐1224‐3p.

### 4.5. miR‐1224‐3p Directly Targeted MKNK2

Potential downstream target genes of miR‐1244‐3p were predicted using four online databases: miRDB (http://mirdb.org; prediction confidence score >70), miRWalk (http://mirwalk.umm.uni-heidelberg.de; score = 1, highest confidence), TargetScanHuman 8.0 (https://www.targetscan.org), and miRTarBase (https://mirtarbase.cuhk.edu.cn/). The above screening identified four candidate target genes: MKNK2, ATF6B, SCD, and ZNF500.

Based on these predictive results, a search of the literature confirmed that MKNK2 is associated with neurological disorders, immune and inflammatory responses, and plays a critical role in the MAPK signaling pathway, prompting its selection for further investigation (Figure 6A). Analysis of peripheral blood RNA by qRT‐PCR in 50 patients with MG and 50 healthy controls revealed that MKNK2 expression was increased in the MG (Figure 6B). Pearson correlation analysis indicated a negative correlation between miR‐1224‐3p and MKNK2 expression, and a positive correlation between hsa_circ_0000313 and MKNK2 expression (Figure 6C). The complementary binding sites of miR‐1224‐3p and MKNK2 are shown in Figure 6A. Knockdown of MKNK2 was performed in Jurkat cells using four siRNAs. Three of these effectively reduced MKNK2 expression, with si‐MKNK2‐1# exhibiting the strongest inhibitory effect (Supporting Information 2: Figure S2B). To validate the hsa_circ_0000313/miR‐1224‐3p/MKNK2 axis, Jurkat cells were transfected with si‐circ_0000313‐3# and miR‐1224‐3p‐mimic, respectively. Subsequent qRT‐PCR and western blot analyses both demonstrated decreased MKNK2 expression (Figure 6D). Both qRT‐PCR and western blot analyses showed that MKNK2 expression was upregulated in the si‐circ_0000313‐3# + miR‐1224‐3p‐inhibitor group, indicating that the miR‐1224‐3p‐inhibitor reversed the downregulatory effect of si‐hsa_circ_0000313‐3# on MKNK2 (Figure 7A). Similarly, both assays demonstrated that the miR‐1224‐3p‐inhibitor reversed the suppression of MKNK2 expression induced by si‐MKNK2‐1# (Figure 7B). The dual‐luciferase reporter assay showed that transfection with the miR‐1224‐3p mimic inhibited the luciferase activity of MKNK2‐3′UTR‐WT, but had no significant effect on that of MKNK2‐3′UTR‐MUT (Figure 7C). Collectively, these results indicated that hsa_circ_0000313 regulates MKNK2 expression through miR‐1224‐3p in MG CD4^+^ T cells.

Figure 6miR‐1224‐3p directly targeted MKNK2. (A) miRDB, miRWalk, TargetScanHuman 8.0, and miRTarBase predicted four potential target mRNAs of miR‐1224‐3p and provided the binding sequences of miR‐1224‐3p with MKNK2. (B) Expression levels of MKNK2 in peripheral blood from 50 MG patients and 50 healthy controls detected by qRT‐PCR. (C) Correlations of both miR‐1224‐3p and hsa_circ_0000313 with MKNK2 were assessed in peripheral blood from 50 MG patients by Pearson’s correlation test. (D) The effects of si‐circ_0000313‐3# and miR‐mimic on MKNK expression were determined by qRT‐PCR and western blot.  ^∗∗^ *p* < 0.01,  ^∗∗∗^ *p* < 0.001, and  ^∗∗∗∗^ *p* < 0.0001.

**Figure:**
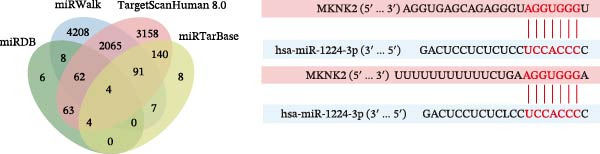
(A)

**Figure:**
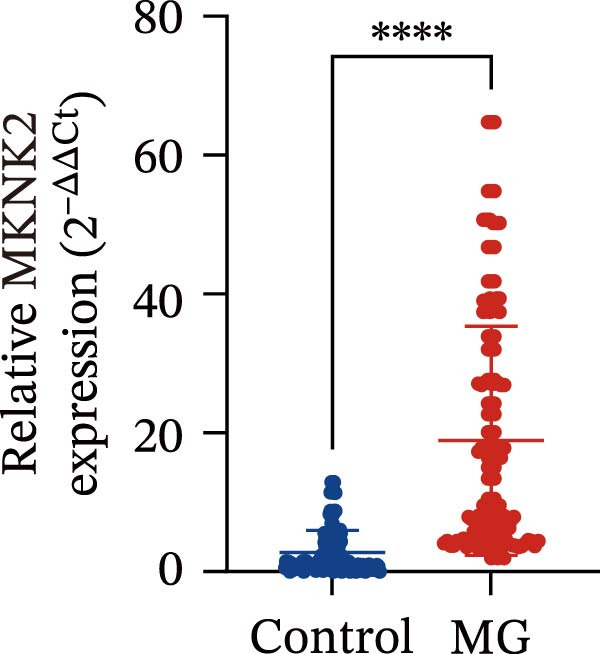
(B)

**Figure:**
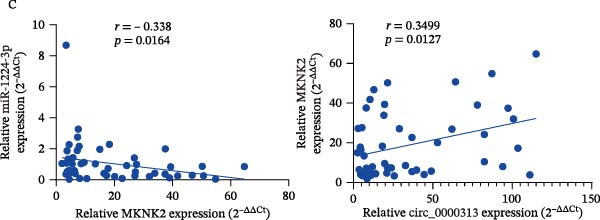
(C)

**Figure:**
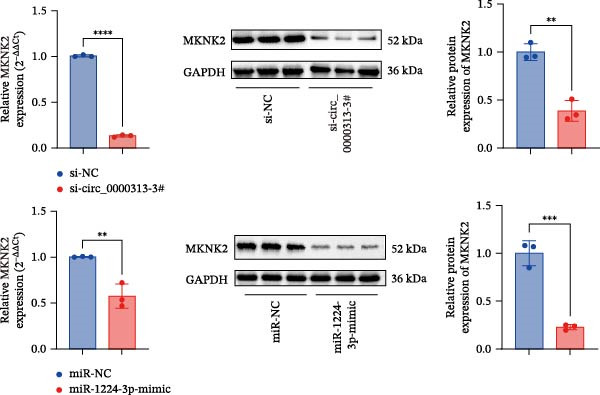
(D)

Figure 7miR‐1224‐3p directly targeted MKNK2. (A, B) The effects of si‐circ_0000313‐3# + miR‐1224‐3p inhibitor and si‐MKNK2‐1# + miR‐1224‐3p inhibitor on MKNK expression were determined by qRT‐PCR and western blot. (C) Dual‐luciferase reporter assay was performed to identify the association of miR‐1224‐3p with MKNK2.  ^∗^ *p* < 0.05,  ^∗∗^ *p* < 0.01,  ^∗∗∗^ *p* < 0.001, and  ^∗∗∗∗^ *p* < 0.0001.

**Figure:**
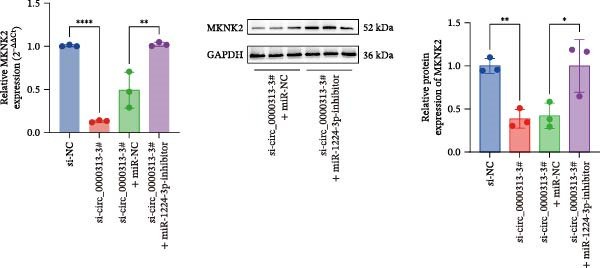
(A)

**Figure:**
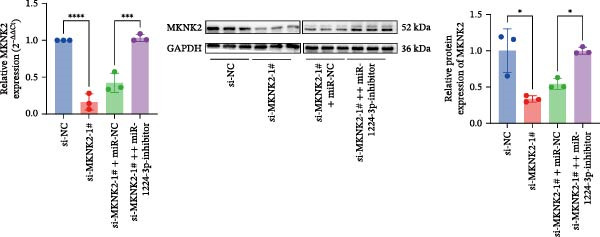
(B)

**Figure:**
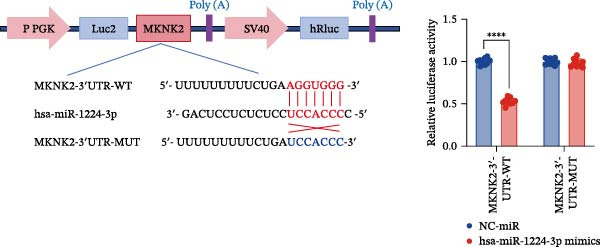
(C)

### 4.6. miR‐1224‐3p Modulated Cellular Behaviors by Regulating MKNK2

We investigated the biological function of MKNK2 in MG. CCK‐8 assays showed that transfection with si‐MKNK2‐1# reduced Jurkat cell proliferation at 48, 72, and 96 h and decreased PCNA protein expression. In contrast, transfection with si‐MKNK2‐1# + miR‐1224‐3p‐inhibitor enhanced cell proliferation and increased PCNA expression at the same time points (Figure 8A).

Figure 8miR‐1224‐3p modulated cellular behaviors by regulating MKNK2. Jurkat cells were divided into si‐NC group, si‐MKNK2‐1# group, si‐MKNK2‐1#+ miR‐NC group, and si‐MKNK2‐1# + miR‐1224‐3p inhibitor group. (A) Cell proliferation was assessed by CCK‐8 assay. The protein level of PCNA was determined by western blot. (B, C) Analysis of the apoptotic cell rate by flow cytometry. The protein levels of Bcl‐2 and Bax were determined by western blot. (D) ELISA for quantifying inflammatory cytokines.  ^∗^ *p* < 0.05,  ^∗∗^ *p* < 0.01,  ^∗∗∗^ *p* < 0.001, and  ^∗∗∗∗^ *p* < 0.0001.

**Figure:**
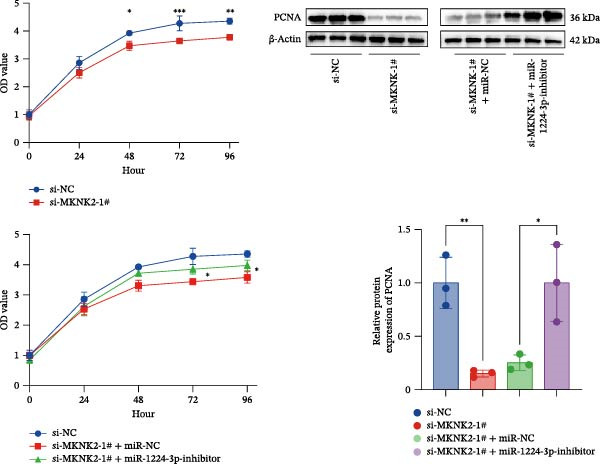
(A)

**Figure:**
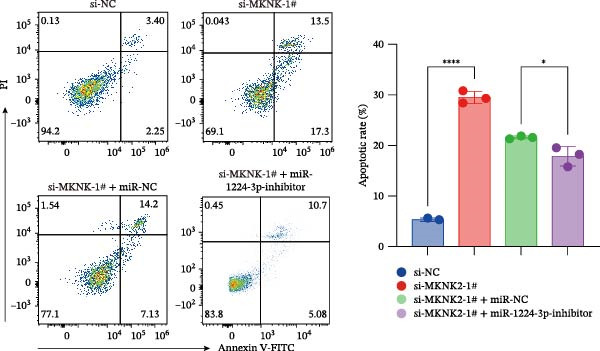
(B)

**Figure:**
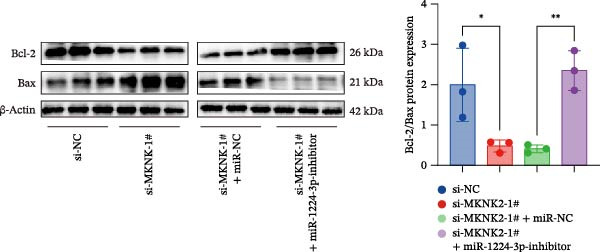
(C)

**Figure:**
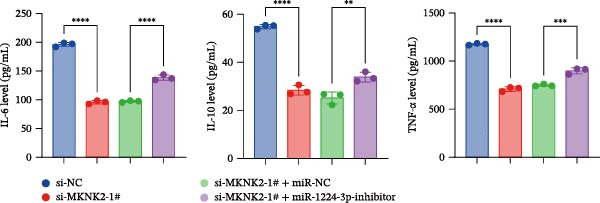
(D)

Flow cytometric analysis showed that transfection with si‐MKNK2‐1# increased the apoptosis rate of Jurkat cells (Figure 8B), decreased Bcl‐2 levels, increased BAX expression, and decreasing the Bcl‐2/BAX ratio (Figure 8C). In contrast, cotransfection with si‐MKNK2‐1# and miR‐1224‐3p‐inhibitor resulted in reduced apoptosis (Figure 8B), elevated Bcl‐2 levels, decreased BAX expression, and increasing the Bcl‐2/BAX ratio (Figure 8C).

ELISA results demonstrated that the secretion levels of TNF‐α, IL‐10, and IL‐6 were significantly downregulated in the si‐MKNK2‐1# group. Transfection with si‐MKNK2‐1# + miR‐1224‐3p‐inhibitor resulted in elevated secretion levels of TNF‐α, IL‐10, and IL‐6 in Jurkat cells (Figure 8D).

These results indicate that MKNK2 and hsa_circ_0000313 produce identical effects on cell proliferation activity, inflammatory cytokine secretion, and apoptosis, all of which oppose the effects of microRNA‐1224‐3p. The miRNA‐1224‐3p inhibitor reversed both the inhibitory effects of si‐MKNK2‐1# on cell proliferation and inflammatory cytokine secretion and its promotive effect on apoptosis. These findings demonstrate that miR‐1224‐3p exerts its function by targeting MKNK2.

### 4.7. hsa_circ_0000313 Regulated the p38 MAPK Signaling Pathway Through the miR‐1224‐3p/MKNK2 Axis

To explore the impact of hsa_circ_0000313 on the p38 MAPK signaling pathway via the miR‐1224‐3p/MKNK2 axis, the phosphorylation level of p38 protein (p‐p38), which is a critical signaling molecule in the p38 MAPK pathway, was measured using western blot analysis (Figure 9A). The si‐circ_0000313‐3#, miR‐1224‐3p‐mimic, and si‐MKNK2‐1# groups demonstrated reduced levels of p‐p38 protein, while the total p38 levels showed no significant differences. The si‐circ_0000313‐3# + miR‐1224‐3p‐inhibitor group exhibited increased levels of p‐p38 protein with no alteration in total p38 levels. Similarly, the si‐MKNK2‐1# + miR‐1224‐3p‐inhibitor group showed elevated p‐p38 without changes in total p38 expression. These results indicate that the miR‐1224‐3p‐inhibitor reverses the downregulation of p38 phosphorylation induced by both si‐circ_0000313‐3# and si‐MKNK2‐1# in the p38 MAPK signaling pathway. Collectively, these findings demonstrate that hsa_circ_0000313 upregulates MKNK2 expression through miR‐1224‐3p, thereby activating the p38 MAPK signaling pathway.

Figure 9hsa_circ_0000313 regulated the p38 MAPK signaling pathway through the miR‐1224‐3p/MKNK2 axis. (A) Jurkat cells were divided into si‐NC group, si‐circ_0000313‐3# group, miR‐NC group, miR‐1224‐3p mimic group, si‐circ_0000313‐3# + miR‐NC group, si‐circ_0000313‐3# + miR‐1224‐3p inhibitor group, si‐MKNK2‐1# group, si‐MKNK2‐1# + miR‐NC group, and si‐MKNK2‐1# + miR‐1224‐3p inhibitor group. The protein levels of p‐p38 and p38 were determined by western blot. (B) Jurkat cells were divided into si‐NC group, si‐circ_0000313‐3# group, si‐circ_0000313‐3# + LPS group. The protein levels of p‐p38 and p38 were determined by western blot.  ^∗∗^ *p* < 0.01 and  ^∗∗∗^ *p* < 0.001.

**Figure:**
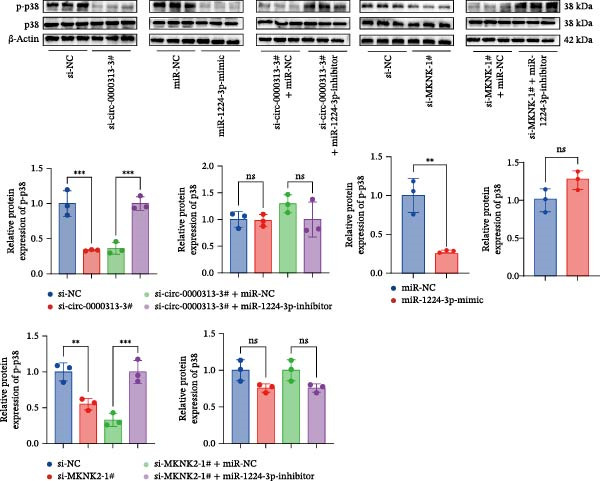
(A)

**Figure:**
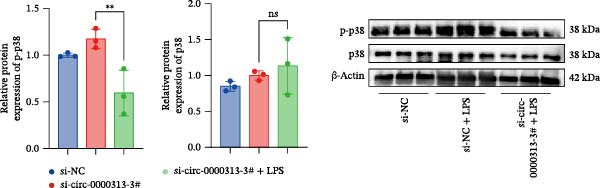
(B)

To further explore the impact of hsa_circ_0000313 on the p38 MAPK signaling pathway, cells were divided into three groups: si‐NC, si‐NC + LPS, and si‐circ_0000313‐3# + LPS. Compared with the si‐NC group, the si‐NC + LPS group exhibited increased levels of p‐p38 protein. Elevated levels of p‐p38 were also observed in the si‐NC + LPS group when compared with the si‐circ_0000313‐3# + LPS group (Figure 9B).

The CCK‐8 assay showed that compared with the si‐NC group, the si‐NC + LPS group exhibited reduced cell proliferation at 24, 48, 72, and 96 h and decreased PCNA protein expression. Furthermore, compared with the si‐NC + LPS group, the si‐circ_0000313‐3# + LPS group showed a further reduction in proliferation at 72 and 96 h and lower PCNA expression (Figure 10A). Flow cytometric analysis revealed that the si‐NC + LPS group exhibited a significantly higher apoptosis rate compared with the si‐NC group (Figure 10B). Accordingly, the levels of Bcl‐2 were decreased and those of BAX were increased in the si‐NC + LPS group compared to the si‐NC group, decreasing the Bcl‐2/BAX ratio (Figure 10C). Compared with the si‐NC + LPS group, the si‐circ_0000313‐3# + LPS group exhibited a further increase in apoptosis (Figure 10B), with corresponding decreases in Bcl‐2 and increases in BAX levels, further decreasing the Bcl‐2/Bax ratio (Figure 10C). ELISA results demonstrated that the secretion of TNF‐α, IL‐10, and IL‐6 was increased in the si‐NC + LPS group compared to the si‐NC group. In contrast, the si‐circ_0000313‐3# + LPS group showed decreased secretion of these cytokines compared to the si‐NC + LPS group (Figure 10D).

Figure 10hsa_circ_0000313 regulated the p38 MAPK signaling pathway through the miR‐1224‐3p/MKNK2 axis. Jurkat cells were divided into si‐NC group, si‐circ_0000313‐3# group, and si‐circ_0000313‐3# + LPS group. (A) Cell proliferation was assessed by CCK‐8 assay. The protein level of PCNA was determined by western blot. (B) Analysis of the apoptotic cell rate by flow cytometry. (C) The protein levels of Bcl‐2 and Bax were determined by western blot. (D) ELISA for quantifying inflammatory cytokines.  ^∗^ *p* < 0.05,  ^∗∗^ *p* < 0.01, and  ^∗∗∗∗^ *p* < 0.0001.

**Figure:**
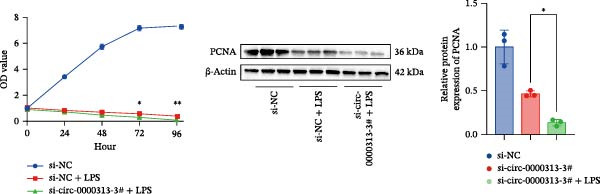
(A)

**Figure:**
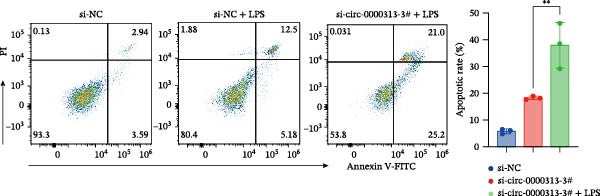
(B)

**Figure:**
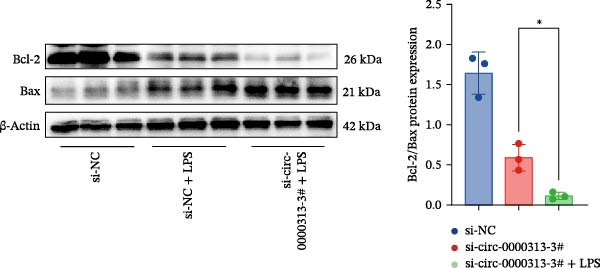
(C)

**Figure:**
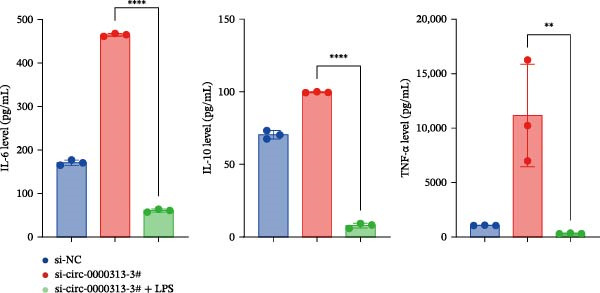
(D)

These results suggest that knockdown of hsa_circ_0000313 inhibited the p38 MAPK signaling pathway in LPS–activated Jurkat cells, thereby suppressing the inflammatory response, inhibiting cell proliferation, and promoting apoptosis.

## 5. Discussion

MG is an autoimmune disorder targeting the neuromuscular junction, characterized by a chronic clinical course and fluctuating muscle weakness symptoms [35]. Its pathological essence involves autoantibody‐mediated impairment of neuromuscular transmission. These autoantibodies are derived from B cells, whereas B cell activation, differentiation, and autoantibody production are largely dependent on help from CD4^+^ T cells. Thus, T cell dysfunction serves as the initiating and central event in the autoimmune response of MG, which represents a key step in the breakdown of immune tolerance and the triggering of autoimmune attack [36]. The standard management of this disease requires long‐term immunosuppressive therapy to control symptoms. However, this prolonged pharmacological strategy carries substantial risks, including disruption of immune homeostasis and induction of serious treatment‐related adverse effects, posing a major clinical challenge [37]. IL‐6, IL‐10, and TNF‐α are pivotal inflammatory cytokines demonstrating elevated secretion in patients with MG, with IL‐10 principally produced by Th2 cells, IL‐6 serving as the critical driver for differentiation of naive CD4^+^ T cells into pathogenic Th17 cells, and TNF‐α promoting IL‐6 release [38–41]. Consequently, there is a pressing imperative to elucidate the pathogenic mechanisms of MG and establish tailored therapeutic strategies for individual patients.

Numerous studies have demonstrated that circRNAs serve as potential biomarkers and are involved in the pathogenesis of multiple autoimmune diseases [32, 42–44]. Our findings demonstrate that hsa_circ_0000313 modulates CD4^+^ T cell activity in MG by sponging miR‐1224‐3p, mediating MKNK2 expression, and acting through the p38 MAPK pathway.

Niu et al. [45] demonstrated that hsa_circRNA_100833 (identified as circFADS2), which is lowly expressed in sepsis, protects lung cells from LPS–induced apoptosis by downregulating miR‐133a. Hong et al. [46] demonstrated that circFADS2, which is upregulated in sepsis, inhibits LPS–induced apoptosis in lung cells by suppressing the maturation of miR‐15a‐5p. circRNA FADS2 plays a pivotal role in multiple pathological conditions. Zhao et al. [47] demonstrated that hsa_circRNA_100833 (identified as circFADS2) is involved in lung cancer pathogenesis. The functional role of circFADS2 has also been observed in colorectal cancer research [48, 49]. Related studies found that hsa_circ_0022387 (identified as circFADS2) was upregulated in rat chondrocytes following LPS treatment, and Li et al. [50] proposed a potential role for it in the regulation of LPS–induced inflammation in chondrocytes. As previously mentioned, infection represents a common trigger for MG exacerbation. hsa_circ_0000313 is derived from the FADS2 host gene. The expression level of FADS2 is significantly associated with key features of the cancer immune microenvironment. Furthermore, FADS2 itself plays a role in regulating immune cell infiltration, the expression of immune regulatory genes, and chemokine network activity [51]. So far, there are no reports on the function or mechanism of hsa_circ_0000313 in MG. Our data demonstrated upregulated expression of hsa_circ_0000313 in patients with MG. Functional verification was conducted in Jurkat cells, and hsa_circ_0000313 was identified as a cytoplasmic circRNA with a circular structure. Knockdown of hsa_circ_0000313 significantly inhibited the proliferation, inflammatory factor secretion, and promoted the apoptosis of Jurkat cells.

Both circRNAs and miRNAs are predominantly localized in the cytoplasm, where cytoplasmic circRNAs exert their biological functions by acting as molecular sponges for miRNAs [52, 53]. Recent studies have identified microRNAs as novel diagnostic biomarkers for MG [54–56]. Zhang et al. [57] demonstrated that miR‐1224‐3p was downregulated in rheumatoid arthritis, thereby affecting disease progression. Circ‐ZNF609 functions as a ceRNA for miR‐1224‐3p to regulate PLK1 expression, mediating the migration and invasion of glioma cells [58]. In the physiological and pathological processes of MG, circRNAs competitively bind to miRNAs and positively regulate mRNA expression through the ceRNA network [54]. KEGG pathway enrichment analysis demonstrated that miR‐1224‐3p is enriched in multiple cancer‐related pathways and functions in GABA ergic synapse, MAPK signaling pathway, neuroactive ligand–receptor interaction, and inflammatory mediator regulation of TRP channels [59]. This study confirmed that hsa_circ_0000313 binds to miR‐1224‐3p, with significant colocalization of both molecules in the cytoplasm. The expression of miR‐1224‐3p was found to be downregulated in patients with MG. Furthermore, hsa_circ_0000313 negatively regulates miR‐1224‐3p, and its effects on Jurkat cell proliferation, inflammatory cytokine secretion, and apoptosis are opposite to those of miR‐1224‐3p. Notably, miR‐1224‐3p can reverse the regulatory effects of hsa_circ_0000313 on these cellular processes, indicating that hsa_circ_0000313 mediates its functions in Jurkat cells through miR‐1224‐3p.

miRNAs inhibit or degrade posttranscriptional mRNAs by targeting their 3′UTRs and participate in multiple regulatory pathways including immune responses, tumorigenesis, organogenesis, cell proliferation, and apoptosis [60, 61]. Predicted targets of miR‐1224‐3p include ATF6B, SCD, MKNK2, and ZNF500, among which MKNK2 has been associated with infection, inflammation, immune escape, and nervous system tumors in related studies [62–64]. A study by Zhang et al. [65] also demonstrated a significant association of MKNK2 with both immune cell infiltration and immune checkpoint expression. Research from Bartish et al. [66] demonstrated that MKNK2 signaling governs the anti‐inflammatory capacity of macrophages through its role as a pivotal node for the selective control of mRNA translation. The selection of MKNK2 from the four bioinformatically predicted targets for detailed mechanistic investigation was based on its strong association with nervous system diseases, immune pathologies, and its key position in the MAPK signaling pathway, designating it as the most disease‐relevant candidate likely to mediate the observed phenotype. Therefore, we selected MKNK2 to validate its targeting relationship with miR‐1224‐3p to elucidate the molecular mechanisms underlying MG progression. MKNK2 is upregulated in patients with MG, and our findings confirm that the hsa_circ_0000313/miR‐1224‐3p/MKNK2 axis is involved in Jurkat cell proliferation and apoptosis. hsa_circ_0000313 and miR‐1224‐3p positively and negatively regulate MKNK2 expression, respectively, while miR‐1224‐3p reverses the suppressive effects of both si‐circ_0000313 and si‐MKNK2 on MKNK2. Knockdown of MKNK2 significantly inhibited the proliferation and inflammatory cytokine secretion of Jurkat cells and promoted their apoptosis. MKNK2 regulates Jurkat cell proliferation, inflammatory cytokine secretion, and apoptosis in a manner consistent with hsa_circ_0000313, but opposite to miR‐1224‐3p, while miR‐1224‐3p reverses these regulatory effects mediated by MKNK2.

Elevated miR‐92a‐2‐5p expression has been found to suppress MKNK2, reduce phosphorylation in the p38 MAPK signaling pathway, and thereby ameliorate oxidative stress damage in cardiomyocytes [67, 68]. MKNK2 is one of two serine/threonine kinases and serves as a substrate for the MAPK pathway [69]. The p38 MAPK pathway is activated by cellular stress and pro‐inflammatory stimuli, stabilizes mRNAs encoding inflammatory proteins including IL‐6 and TNF‐α [70]; and these findings are consistent with our results. Extracellular lipopolysaccharide from gram‐negative bacteria plays a critical role in innate immune responses and exacerbation of inflammation. LPS activates the TLR4 signaling pathway, subsequently engages p38 MAPK, and ultimately induces immune and inflammatory responses [71], while Zheng et al. [72] demonstrated the role of p38 MAPK in LPS–induced inflammation, and knockdown of lncRNA‐PVT1 ameliorates LPS–induced macrophage inflammation by suppressing the p38 MAPK signaling pathway and reducing IL‐1β and TNF‐α mRNA expression.

We verified the involvement of the hsa_circ_0000313/miR‐1224‐3p/MKNK2 axis in p38MAPK expression, respectively. We established an inflammatory model where LPS stimulation of Jurkat cells promoted inflammatory cytokine secretion and apoptosis while inhibiting cell proliferation. LPS stimulation of Jurkat cells increased p38 MAPK pathway activity. Conversely, hsa_circ_0000313 knockdown in LPS–stimulated Jurkat cells significantly suppressed p38 MAPK pathway activation, inflammatory cytokine secretion, cell proliferation, and promoted apoptosis. Knockdown of hsa_circ_0002783 may suppress the MAPK signaling pathway, ameliorating LPS–induced inflammation in Jurkat cells. This study elucidates the role and mechanistic insights of hsa_circ_0000313 in LPS–induced inflammatory responses in Jurkat cells, providing a promising novel therapeutic target for molecular‐level intervention and regulation of inflammation in patients with MG.

Our study has limitations: while we detected elevated expression of hsa_circ_0000313 in PBMCs from MG patients, we did not examine its expression patterns in specific immune cell subtypes. Future investigations should include animal experiments to verify the involvement of the hsa_circ_0000313/miR‐1224‐3p/MKNK2 axis in the p38 MAPK signaling pathway. Due to the limited sample size in this exploratory study, we will in the future conduct research with an expanded patient cohort.

## 6. Conclusion

In conclusion, our results demonstrate that hsa_circ_0000313 is upregulated in MG patients, and by sponging miR‐1224‐3p, it increases MKNK2 expression and activates the p38 MAPK pathway, thereby affecting Jurkat cell proliferation, apoptosis, and inflammatory cytokine secretion. In LPS–induced Jurkat cells, knockdown of hsa_circ_0000313 ameliorated inflammation, potentially through suppression of p38 MAPK signaling pathway activation.

## Funding

This work was funded by the National Natural Science Foundation of China (Grants 82171396 and 82271434), the Heilongjiang Provincial Natural Science Foundation Project – Outstanding Youth (Grant YQ2023H011), and the Heilongjiang Chunyan Youth Science and Technology Talent team project (Grant CYQN24009).

## Conflicts of Interest

The authors declare no conflicts of interest.

## Supporting Information

Additional supporting information can be found online in the Supporting Information section.

## Supporting information


**Supporting Information 1** By applying the thresholds of |log_2_FC| > 0.5 and *p*‐value <0.05 to the circRNA microarray sequencing dataset, a list of candidate differentially expressed circRNAs warranting further investigation was generated, as detailed in Table S1. Detailed information on the groups for dual‐luciferase transfection is presented in Tables S2 and S3.

**Supporting Information 2** Figure S1. The knockdown efficiency of siRNA targeting was assessed. (A) Knockdown efficiency of three siRNAs targeting hsa_circ_0000313 as validated by qRT‐PCR. (B) Knockdown efficiency of three siRNAs targeting MKNK2 as validated by qRT‐PCR.

## Data Availability

The data that support the findings of this study are available upon request from the corresponding author. The data are not publicly available due to privacy or ethical restrictions.
