# Supplementary material for: Hsa_circ_0000313/miR‐1224‐3p/MKNK2 Axis Modulates CD4+ T Cells by Activating p38 MAPK Signaling in Myasthenia Gravis
Source: Mediators Inflamm. 2026 Mar 20;2026:2877539. doi: 10.1155/mi/2877539 (PMC13140341; doi:10.1155/mi/2877539)
Supplement: Supplementary file 1 — Supporting Information 1 By applying the thresholds of |log2FC| > 0.5 and p‐value <0.05 to the circRNA microarray sequencing dataset, a list of candidate differentially expressed circRNAs warranting further investigation was generated, as detailed in Table S1. Detailed information on the groups for dual‐luciferase transfection is presented in Tables S2 and S3. [file MI-2026-2877539-s001.zip › Research Guidelines checklist.docx]

|  | **Section & Topic** | **No** | **Item** | **Reported on page #** |
| --- | --- | --- | --- | --- |
|  |  |  |  |  |
|  | **TITLE OR ABSTRACT** |  |  |  |
|  |  | **1** | Identification as a study of diagnostic accuracy using at least one measure of accuracy  (such as sensitivity, specificity, predictive values, or AUC) | Line 1 /Page 1 |
|  | **ABSTRACT** |  |  |  |
|  |  | **2** | Structured summary of study design, methods, results, and conclusions  (for specific guidance, see STARD for Abstracts) | Line 19-46 /Page 1-2 |
|  | **INTRODUCTION** |  |  |  |
|  |  | **3** | Scientific and clinical background, including the intended use and clinical role of the index test | Line 48-78 /Page 2-3 |
|  |  | **4** | Study objectives and hypotheses | Line 75-115 /Page 3-4 |
|  | **METHODS** |  |  |  |
|  | *Study design* | **5** | Whether data collection was planned before the index test and reference standard  were performed (prospective study) or after (retrospective study) | Line 118-120 /Page 5 |
|  | *Participants* | **6** | Eligibility criteria | Line 120-129 /Page 5 |
|  |  | **7** | On what basis potentially eligible participants were identified  (such as symptoms, results from previous tests, inclusion in registry) | Line 120-129 /Page 5 |
|  |  | **8** | Where and when potentially eligible participants were identified (setting, location and dates) | Line 120-129 /Page 5 |
|  |  | **9** | Whether participants formed a consecutive, random or convenience series | Line 120-129 /Page 5 |
|  | *Test methods* | **10a** | Index test, in sufficient detail to allow replication | Not Applicable |
|  |  | **10b** | Reference standard, in sufficient detail to allow replication | Line 120-121 /Page 5 |
|  |  | **11** | Rationale for choosing the reference standard (if alternatives exist) | Line 117-129 /Page 5 |
|  |  | **12a** | Definition of and rationale for test positivity cut-offs or result categories  of the index test, distinguishing pre-specified from exploratory | Line 117-129 /Page 5 |
|  |  | **12b** | Definition of and rationale for test positivity cut-offs or result categories  of the reference standard, distinguishing pre-specified from exploratory | Line 117-129 /Page 5 |
|  |  | **13a** | Whether clinical information and reference standard results were available  to the performers/readers of the index test | Line 117-129 /Page 5 |
|  |  | **13b** | Whether clinical information and index test results were available  to the assessors of the reference standard | Line 130-142 /Page 5 |
|  | *Analysis* | **14** | Methods for estimating or comparing measures of diagnostic accuracy | Line 231-239 /Page 8-9 |
|  |  | **15** | How indeterminate index test or reference standard results were handled | Line 116-230 /Page 5-8 |
|  |  | **16** | How missing data on the index test and reference standard were handled | Line 116-230 /Page 5-8 |
|  |  | **17** | Any analyses of variability in diagnostic accuracy, distinguishing pre-specified from exploratory | Line 116-230 /Page 5-8 |
|  |  | **18** | Intended sample size and how it was determined | Line 116-230 /Page 5-8 |
|  | **RESULTS** |  |  |  |
|  | *Participants* | **19** | Flow of participants, using a diagram | Not Applicable |
|  |  | **20** | Baseline demographic and clinical characteristics of participants | Can be provided later |
|  |  | **21a** | Distribution of severity of disease in those with the target condition | Line 117-129 /Page 5 |
|  |  | **21b** | Distribution of alternative diagnoses in those without the target condition | Can be provided later |
|  |  | **22** | Time interval and any clinical interventions between index test and reference standard | Not Applicable |
|  | *Test results* | **23** | Cross tabulation of the index test results (or their distribution)  by the results of the reference standard | Line 240-390 /Page 9-14 |
|  |  | **24** | Estimates of diagnostic accuracy and their precision (such as 95% confidence intervals) | Line 231-239 /Page 8-9 |
|  |  | **25** | Any adverse events from performing the index test or the reference standard | Not Applicable |
|  | **DISCUSSION** |  |  |  |
|  |  | **26** | Study limitations, including sources of potential bias, statistical uncertainty, and generalisability | Line 490-500 /Page 18 |
|  |  | **27** | Implications for practice, including the intended use and clinical role of the index test | Line 484-495 /Page 17-18 |
|  | **OTHER INFORMATION** |  |  |  |
|  |  | **28** | Registration number and name of registry | Line 125-129 /Page 5 |
|  |  | **29** | Where the full study protocol can be accessed | Not Applicable |
|  |  | **30** | Sources of funding and other support; role of funders | Line 509-513 /Page 18 |
|  |  |  |  |  |
