# Supplementary material for: Hsa_circ_0000313/miR‐1224‐3p/MKNK2 Axis Modulates CD4+ T Cells by Activating p38 MAPK Signaling in Myasthenia Gravis
Source: Mediators Inflamm. 2026 Mar 20;2026:2877539. doi: 10.1155/mi/2877539 (PMC13140341; doi:10.1155/mi/2877539)
Supplement: Supplementary file 1 — Supporting Information 1 By applying the thresholds of |log2FC| > 0.5 and p‐value <0.05 to the circRNA microarray sequencing dataset, a list of candidate differentially expressed circRNAs warranting further investigation was generated, as detailed in Table S1. Detailed information on the groups for dual‐luciferase transfection is presented in Tables S2 and S3. [file MI-2026-2877539-s001.zip › Supplementary Table 1 2 3.docx]

Supplementary Table 1. Differentially expressed circRNAs

| circRNA | circBase | GeneSymbol | | chrom | | circRNA type | circRNA length | Regulation |
| --- | --- | --- | --- | --- | --- | --- | --- | --- |
| hsa_circRNA_062400 | hsa_circ_0062400 | CRKL | chr22 | | exonic | | 5325 | up |
| hsa_circRNA_404827 | / | SCART1 | chr10 | | exonic | | 2977 | up |
| hsa_circRNA_000313 | hsa_circ_0000313 | FADS2 | chr11 | | overlapping | | 318 | up |
| hsa_circRNA_004183 | hsa_circ_0004183 | FRMD4A | chr10 | | exonic | | 949 | up |
| hsa_circRNA_101525 | hsa_circ_0035381 | PIGB | chr15 | | exonic | | 601 | up |
| hsa_circRNA_045234 | hsa_circ_0045234 | DDX42 | chr17 | | exonic | | 225 | up |
| hsa_circRNA_405788 | / | CADM4 | chr19 | | exonic | | 993 | up |
| hsa_circRNA_403482 | / | RBM22 | chr5 | | exonic | | 234 | up |
| hsa_circRNA_092465 | hsa_circ_0000911 | GTPBP3 | chr19 | | antisense | | 531 | down |
| hsa_circRNA_039626 | hsa_circ_0039626 | CNOT1 | chr16 | | exonic | | 4474 | down |
| hsa_circRNA_000680 | hsa_circ_0000680 | IQCK | chr16 | | exonic | | 229 | down |
| hsa_circRNA_014551 | hsa_circ_0014551 | ASH1L | chr1 | | exonic | | 490 | down |
| hsa_circRNA_046669 | hsa_circ_0046669 | THOC1 | chr18 | | exonic | | 626 | down |
| hsa_circRNA_000881 | hsa_circ_0000788 | MSI2 | chr17 | | intronic | | 246 | down |
| hsa_circRNA_104387 | hsa_circ_0080425 | WBSCR17 | chr7 | | exonic | | 373 | down |
| hsa_circRNA_404935 | / | ZBTB16 | chr11 | | exonic | | 1358 | down |
| hsa_circRNA_104940 | hsa_circ_0089153 | NUP214 | chr11 | | exonic | | 1102 | down |
| hsa_circRNA_100486 | hsa_circ_0001958 | PCNXL2 | chr1 | | exonic | | 582 | down |
| hsa_circRNA_100147 | hsa_circ_0004240 | EIF3I | chr1 | | exonic | | 275 | down |
| hsa_circRNA_102445 | hsa_circ_0004552 | CARM1 | chr19 | | exonic | | 338 | down |
| hsa_circRNA_004561 | hsa_circ_0004561 | TRIQK | chr8 | | intronic | | 12646 | down |
| hsa_circRNA_081481 | hsa_circ_0081481 | FBXO24 | chr7 | | exonic | | 236 | down |
| hsa_circRNA_400185 | / | ZNF362 | chr1 | | exonic | | 870 | down |
| hsa_circRNA_000740 | hsa_circ_0001579 | ATXN1 | chr6 | | intronic | | 189 | down |
| hsa_circRNA_004509 | hsa_circ_0004509 | DTWD2 | chr5 | | exonic | | 379 | down |
| hsa_circRNA_006526 | hsa_circ_0006526 | ROCK2 | chr2 | | exonic | | 183 | down |
| hsa_circRNA_048764 | hsa_circ_0048764 | RPL36 | chr19 | | exonic | | 537 | down |
| hsa_circRNA_059665 | hsa_circ_0059665 | ABHD12 | chr20 | | exonic | | 445 | down |

Supplementary Table 2. Transfection groups for the Dual-Luciferase reporter Assay

| Group | Luciferase reporter vector | miR |
| --- | --- | --- |
| 1 | hsa_circ_0000313-WT | miR-NC |
| 2 | hsa_circ_0000313-WT | miR-1224-3p-mimics |
| 3 | hsa_circ_0000313-MUT | miR-NC |
| 4 | hsa_circ_0000313-MUT | miR-1224-3p-mimics |

Supplementary Table 3. Transfection groups for the Dual-Luciferase reporter Assay

| Group | Luciferase reporter vector | miR |
| --- | --- | --- |
| 1 | MKNK2-3'UTR -WT | miR-NC |
| 2 | MKNK2-3'UTR -WT | miR-1224-3p-mimics |
| 3 | MKNK2-3'UTR -MUT | miR-NC |
| 4 | MKNK2-3'UTR -MUT | miR-1224-3p-mimics |
