# Supplementary figures and images for: Hsa_circ_0000313/miR‐1224‐3p/MKNK2 Axis Modulates CD4+ T Cells by Activating p38 MAPK Signaling in Myasthenia Gravis
Source: Mediators Inflamm. 2026 Mar 20;2026:2877539. doi: 10.1155/mi/2877539 (PMC13140341; doi:10.1155/mi/2877539)

**A**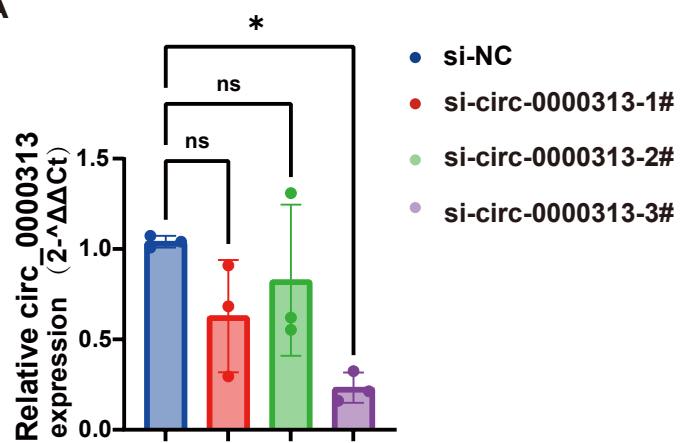**B**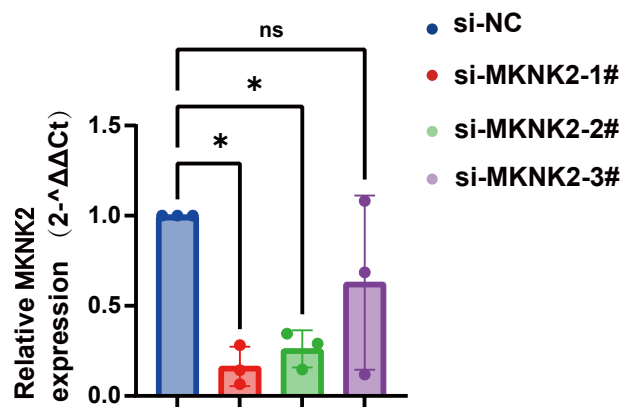

Supplement: Supplementary file 2 — Supporting Information 2 Figure S1. The knockdown efficiency of siRNA targeting was assessed. (A) Knockdown efficiency of three siRNAs targeting hsa_circ_0000313 as validated by qRT‐PCR. (B) Knockdown efficiency of three siRNAs targeting MKNK2 as validated by qRT‐PCR. [file MI-2026-2877539-s002.pdf]
